# A Bio-Inspired Polarization Sensor with High Outdoor Accuracy and Central-Symmetry Calibration Method with Integrating Sphere

**DOI:** 10.3390/s19163448

**Published:** 2019-08-07

**Authors:** Yinlong Wang, Jinkui Chu, Ran Zhang, Jinshan Li, Xiaoqing Guo, Muyin Lin

**Affiliations:** Key Laboratory for Micro/Nano Technology and System of Liaoning Province, Dalian University of Technology, Dalian 116024, China

**Keywords:** navigation sensor, scattered polarization skylight, calibration, outdoor accuracy, integrating sphere

## Abstract

A bio-inspired polarization sensor with lenses for navigation was evaluated in this study. Two new calibration methods are introduced, referred to as “central-symmetry calibration” (with an integrating sphere) and “noncontinuous calibration”. A comparison between the indoor calibration results obtained from different calibration methods shows that the two proposed calibration methods are more effective. The central-symmetry calibration method optimized the nonconstant calibration voltage deviations, caused by the off-axis feature of the integrating sphere, to be constant values which can be calibrated easily. The section algorithm proposed previously showed no experimental advantages until the central-symmetry calibration method was proposed. The outdoor experimental results indicated that the indoor calibration parameters did not perform very well in practice outdoor conditions. To establish the reason, four types of calibration parameters were analyzed using the replacement method. It can be concluded that three types can be easily calibrated or affect the sensor accuracy slightly. However, before the sensor is used outdoors every time, the last type must be replaced with the corresponding outdoor parameter, and the calculation needs a precise rotary table. This parameter, which is mainly affected by the spectrum of incident light, is the main factor determining the sensor accuracy. After calibration, the sensor reaches an indoor accuracy of ±0.009° and a static outdoor accuracy of ±0.05° under clear sky conditions. The dynamic outdoor experiment shows a ±0.5° heading deviation between the polarization sensor and the inertial navigation system with a ±0.06° angular accuracy.

## 1. Introduction

Polarization vision, the ability of some animals to detect the oscillation plane of an electric-field vector of light, has inspired scientists for decades. In 2004, Cochran reported that migrating songbirds recalibrate their internal magnetic compass daily from twilight polarization cues [1]. In 2006, Muheim reported that polarized light cues, in general, underlie the compass calibration of migratory songbirds [2]. The dynamic polarization vision in mantis shrimps was investigated in 2016 [3]. Furthermore, polarization cues also play an important role in homing ants [4]. Polarization navigation based on polarization cues (i.e., the scattered skylight) is an important application of polarization vision. Its advantages are the anti-interference and lack of error accumulations.

A mechanism for bio-inspired polarization sensors for navigation was proposed in [5], and a study of angle measurements using an optoelectronic model to emulate the polarization-sensitive compound eyes of insects was proposed in [6]. Bio-sensors for navigation [7] attempt to imitate the orthogonal structure of insects’ compound eyes [8], which can improve sensor resolution. In 2008, an outdoor accuracy of ±0.2° was obtained using a normalization process for the final polarization angle [7]. In 2014, both a new angle-computation method and a new calibration method were proposed [9]. In 2016, the Non-dominated Sorting Genetic Algorithm- II (NSGA-II) [10] was used to minimize both angle of polarization (AOP) residuals and degree of linear polarization (DOLP) dispersions, which resulted in ±0.2° indoor accuracy. In addition, Xu obtained an outdoor accuracy [11] of ±0.2° in the same year. In 2018, a bionic polarization-navigation sensor based on a polarizing beam-splitter [12] reached ±0.42° outdoor accuracy after calibration. The abovementioned sensors can determine polarization angles in real-time. A sensor, which can measure ultraviolet (UV) light, achieved an accuracy of ±0.3° in clear sky in 2017 [13]; however, it requires scanning. The following measurement offers a median angular error of 0.39° in 2019 [14], and this sensor was used on a six-legged walking robot in an outdoor environment in 2019 [15]. By analyzing the multi-source errors, in indoor experiments in 2019 the unscented Kalman filter (UKF) and the extended Kalman filter (EKF) calibration methods [16] achieved accuracies of 0.0459° and 0.0348°, respectively. In conclusion, after a decade of research, bio-inspired sensors can now perform both short- and long-range navigation tasks well, with just a few pixels [17]. However, the currently achieved outdoor accuracy, between ±0.2° and ±0.42°, is not very high.

When only one polarization-navigation bio-sensor is used, only the orientation information can be obtained. If more sensors are used to measure the scattered skylight, which can be described using the Rayleigh scattered model [18], more complex tasks can be performed. A novel autonomous real-time position-determining method, which uses both polarized light and the geomagnetic field, was reported in 2015 [19]. An orthogonal vector algorithm, used to obtain navigation information, can avail the advantage of redundancy of the Rayleigh model [20]. The polarization-navigation bio-sensor can also help to determine the attitude of a device [21].

Imaging polarimetry is another research field of polarization navigation. It is reported that the Vikings possibly used the polarization patterns of skylight for solving challenging navigation tasks, such as sailing to Greenland from Norway. The authors determined the accuracy [22] of hypothetical sky-polarimetric Viking navigation for 1080 different sky conditions that were characterized by parameters such as solar elevation and cloudiness [23]. A simulation based on a large amount of actual data [23], obtained using imaging polarimetry, shows that Viking sailors could have travelled to Greenland from Norway using polarization information [24]. Furthermore, the angle algorithm has also been improved. An angle algorithm using the Hough transform was proposed in 2015 [25], and outdoor accuracy became 0.37° before any error correction. A novel camera-based bio-inspired polarization navigation sensor was proposed in 2016 [26], and “80% of AOP deviations are less than 2° and 40% less than 1°”. A real-time polarization imaging algorithm was proposed in 2017 [27] that has a refresh rate of about 33 Hz. The angles for skylight polarization, derived using the digital images of sky under various conditions, were studied in 2017 [28]. An integrated polarized skylight sensor and Micro Inertial Measurement Unit (MIMU) with a metric map for urban ground navigation were proposed in 2018 [29].

After comparing the two types of devices described above, we found that imaging polarimetry can reach moderate outdoor polarization-angle accuracy and a lower refresh rate than conventional bio-sensors [5,7,9,12]. Bio-inspired sensors do not have a very high outdoor accuracy. In a practical application, complex tasks such as determining the global position or the attitude of a device require high polarization-angle accuracy for several observed directions, not just high accuracy in one direction. This paper focused on improving the outdoor accuracy. As a result, the relevant devices can perform better in complex navigation tasks. In essence, a bio-sensor can be regarded as one pixel of an imaging system. Therefore, its calibration result is meaningful for the development of an imaging system.

Calibration is necessary to ensure a high accuracy. During the calibration, a light source that can provide uniform light is needed because of the operating principle of a polarization sensor. An integrating sphere is suitable for measuring optical irradiation. It functions based on the radiation exchange within an enclosure of diffuse surfaces. The tested sensor is often placed coaxial with the sphere, but at some distance away from the port of integrating sphere source. The uniformity is 100% at the plane of port. However, it is almost impossible to position the sensor accurately, because the linear-polarizing laminated films which generate linear polarization light are located at the port, and the sensor has a complex inner structure. Therefore, the measured sensor is often placed at some distance away from the port of integrating sphere, thus decreasing uniformity. This effect leads to systematic errors. To weaken this effect, both the sphere and sensor are generally positioned coaxial. However, in practice, there is always a slight off-center distance. As a result, the section algorithm [30], proposed by us in 2012 to use the redundancy of sensor’s light path when detecting the linear polarization light, shows no advantage in indoor calibration experiments. Both the problem of a slight off-center distance and the unavailability of section algorithm are solved in this paper.

In addition, in this study, a polarization bio-sensor with plano-convex lenses was constructed. Because we found that there is no lens in [5,7,9,14], the imitation of compound eyes of insects is not very good. The accuracy limit, determined by a 16-bit analog-to-digital converter (ADC), was calculated in our simulation for different degrees of polarization of incident light.

We also studied and improved the calibration method by exploring two new calibration techniques. To increase the light uniformity decreased by the slight off-center distance when using an integrating sphere, we introduce, what we named as, the “central-symmetry method”. The second calibration technique is the “noncontinuous method”, which is an extension of the section algorithm and serves to further improve the calibration. Subsequently, indoor calibration experiments were performed and compared, and the effect of different calibration parameters on the accuracy was analyzed. Finally, outdoor experiments were performed, and the calibration results were studied using the replacement method.

Table 1 includes the main mathematical variables as below.

## 2. Bio-Sensor for Navigation

### 2.1. Structure

**Figure 1:**
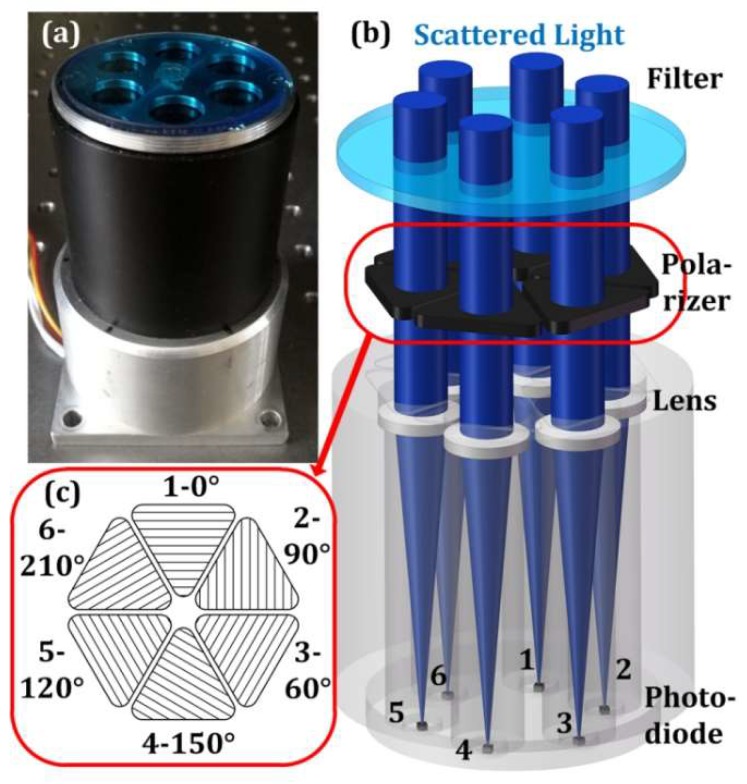
Photograph and schematic of bio-inspired polarization sensor. (**a**) Photograph of sensor; (**b**) light path of scattered light; (**c**) directions of the six polarizers.

The sensor proposed in this paper mainly consisted of a blue filter, six linear-polarizing laminated films, six plano-convex lenses, six photodiodes, and an electronic circuit (see Figure 1a). The height of the sensor is 100 mm and the diameter is 63 mm. The focal length of the plano-convex lens is 50 mm. The diameter is 0.5 inch (12.7 mm). The photodiode is a ceramic package photodiode. Its spectral response range is 320 to 730 nm. The pixel of the photodiode is 1.3 mm × 1.3 mm. The electronic circuit mainly contains a MSP430 with four 16-bit ADCs inside. The refresh rate of the sensor is 150 Hz. There are six light paths that share the same blue filter. In each path, there is a linear-polarizing laminated film (i.e., polarizer), a plano-convex lens, and a photodiode (see Figure 1b). The plano-convex can narrow the field of view and shield stray light, such as direct sunlight. Inspired by insects, six direct-current signals, which are derived from six photodiodes, are divided into three groups. In each group, the two main polarization directions of linear-polarizing films are orthogonal, and the two current signals act as the inputs to a logarithmic amplifier, which has an exponential response to the sunlight that varies from 0.0000146 to 14.64 W/m^2^ when the elevation varies from –6° to 15°. The six main polarization angles are 0° (the reference direction), 90°, 60°, 150°, 120°, and 210° (see Figure 1c). The corresponding photodiode numbers are 1, 2, 3, 4, 5, and 6.

### 2.2. Polarization Angle Calculation

**Figure 2:**
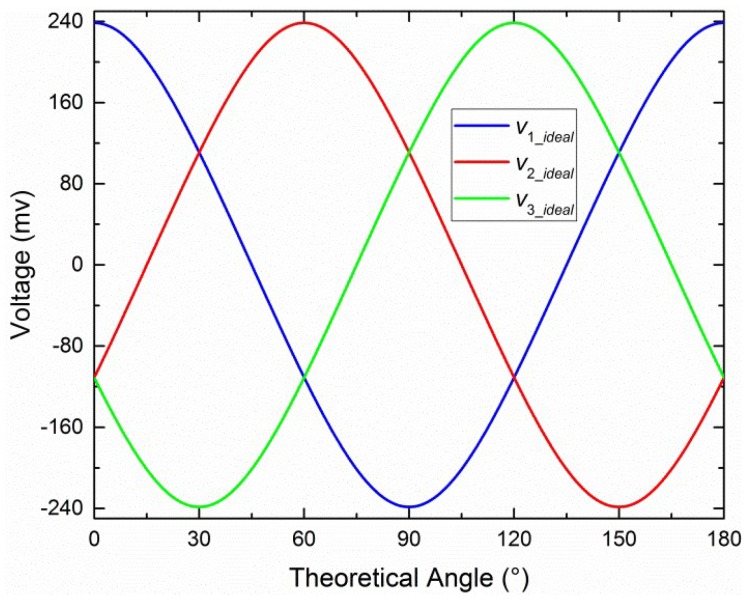
Values for *V*_1_*ideal*_, *V*_2_*ideal*_, and *V*_3_*ideal*_ when *d* = 0.5.

The outputs of the three groups [7] are direct voltages. *V*_1_*ideal*_, *V*_2_*ideal*_, and *V*_3_*ideal*_ are the ideal outputs of photodiodes 1 and 2, 3 and 4, 5 and 6, respectively. They can be described as follows:

(1) $$\begin{array}{l} V_{1{\_}_{ideal}}=0.5\log\frac{1+d\cos(2\theta )}{1-d\cos(2\theta )}\\ V_{2{\_}_{ideal}}=0.5\log\frac{1+d\cos(2\theta -120{}^{\circ})}{1-d\cos(2\theta -120{}^{\circ})}\\ V_{3\_ideal}=0.5\log\frac{1+d\cos(2\theta +120{}^{\circ})}{1-d\cos(2\theta +120{}^{\circ})} \end{array}$$

where “0.5” is the parameter of logarithmic amplifier; *d* is the (dimensionless) polarization degree of incident light. The output of sensor, *θ*, is the angle between the reference direction and the main polarization direction of incident light. The values *d* = 0.5 and *θ* = [0°, 180°] with a 0.1° interval for Equation (1) are shown in Figure 2.

We can obtain three equal outputs (*θ*_12_, *θ*_13_, *θ*_23_) simultaneously. These are derived from *V*_1_*ideal*_ and *V*_2_*ideal*_, *V*_1_*ideal*_ and *V*_3_*ideal*_, *V*_2_*ideal*_ and *V*_3_*ideal*_, respectively.

(2) $$\theta_{12}=\frac{1}{2}\arctan\frac{\left(A-1\right)\left(B+1\right)\cos(120{}^{\circ})-\left(B-1\right)\left(A+1\right)\cos(0{}^{\circ})}{\left(A-1\right)\left(B+1\right)\sin(120{}^{\circ})-\left(B-1\right)\left(A+1\right)\sin(0{}^{\circ})},$$

where *A* = 10^2*V*^^1_*ideal*^ and *B* = 10^2*V*^^2_*ideal*^. In Equation (2), the entire range for *θ*_12_ is 90°. However, this value should be 180° for linear polarized light. Hence, a method is needed to extend the range. Each *θ*_12_ has a corresponding *d*_12_. If the sign of *d*_12_ is negative or positive, then the output angle for *V*_1_*ideal*_ and *V*_2_*ideal*_ is *θ*_12_+90° or *θ*_12_ and the final degree of polarization is −*d*_12_ or *d*_12_. In other words, the sign of *d*_12_ is used to extend the range to 180°. In a similar manner, we can obtain *θ*_13_ and *θ*_23_.

### 2.3. Section Algorithm

Three groups of outputs can be obtained, because there are three equations and only two unknown variables. The section algorithm [30] is proposed to use the redundancy of the sensor’s light path when detecting the linear polarization light. The basic principle is to avoid using *V*, for which the corresponding derivative is 0.

**Figure 3:**
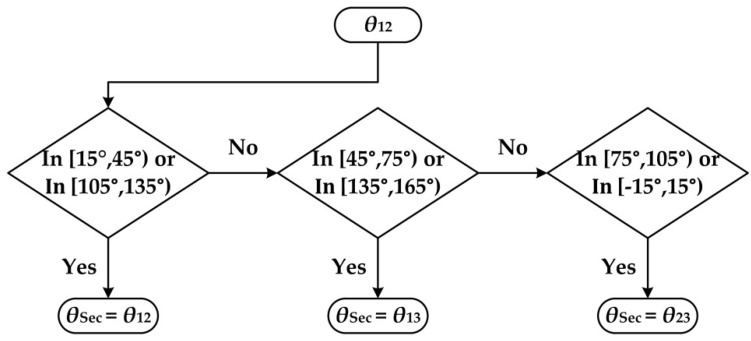
Functional diagram of the section algorithm.

The derivatives are 0 when the denominators in Equation (1) are minimal. In this case, the electrical noise produces large errors. For each *V*, there are two bad points, the derivatives of which are 0. Then, there are six such bad points for our sensor. Therefore, we obtain six sections by averaging the range (0°–180°). The middle point of each section corresponds with the bad point. For each section, *V* with a bad point is excluded and the other two remaining *V* are used to calculate the final output *θ*_sec_. In this section algorithm, when *θ* is in the different section, the corresponding *θ* is considered as the final output. The functional diagram is shown in Figure 3. *θ*_12_ is used to determine the selected section in this paper. For instance, if we assume that *θ*_12_ is 60°, *θ*_13_ is 60.1°, and *θ*_23_ is 60.2°. Then, because *θ*_12_ lies within the section [45°, 75°), the final output *θ*_sec_ is *θ*_13_ (60.1°), and *θ*_23_ is meaningless. More details can be seen in Section 4.4 and Section 5.1.

### 2.4. Accuracy Limit for Different d Values

The accuracy limit for the sensor depends on the 16-bit ADC as well as the degree of polarization *d*. The digital output of the 16-bit AD is from −32,767 to +32,767. In our simulation, aimed at calculating the best theoretical accuracy determined by the integer effect, the voltages can be described as follows:

(3) $$V_{i\_AD}=round\;(V_{i}/V_{ref}\times 2^{15})\times V_{ref}/2^{15},$$

where *V_i_AD_* is rounded to the nearest integer by the rule known as round half up and *i* is 1, 2, or 3. Because *V_i___AD_* includes both positive and negative values, the power of 2 is 15 and not 16 for the 16-bit AD. *V_ref_* is the reference voltage for the logarithmic amplifier, and its value is 600 mV in this paper.

**Figure 4:**
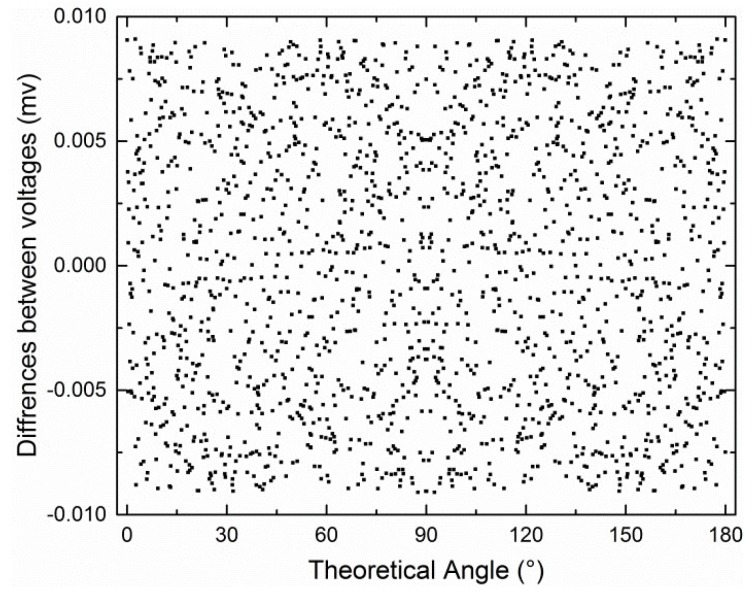
Difference between *V*_1_*ideal*_ and *V*_1_*AD*_ when *d* = 0.5.

The difference between *V*_1_*ideal*_ and *V*_1*_AD*_ is shown in Figure 4. The horizontal axis represents the theoretical values of linear angle with a 0.1° interval without any errors. The differences between *V*_2_*ideal*_ and *V*_2*_AD*_ and between *V*_3_*ideal*_ and *V*_3*_AD*_ are not shown because they look similar.

**Figure 5:**
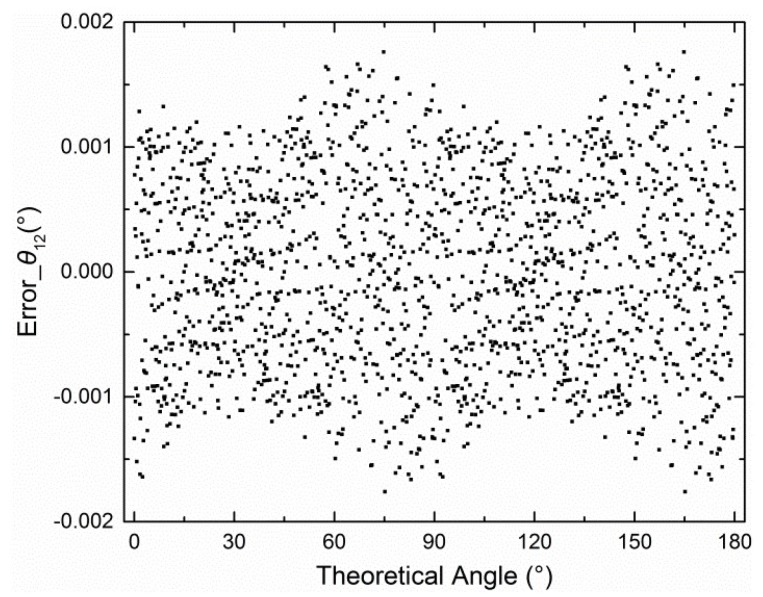
Errors for *θ*_12_ when *d* = 0.5.

The error curves were also similar. Therefore, only the error curve derived from *V*_1*_AD*_ and *V*_2*_AD*_ is shown in Figure 5.

**Figure 6:**
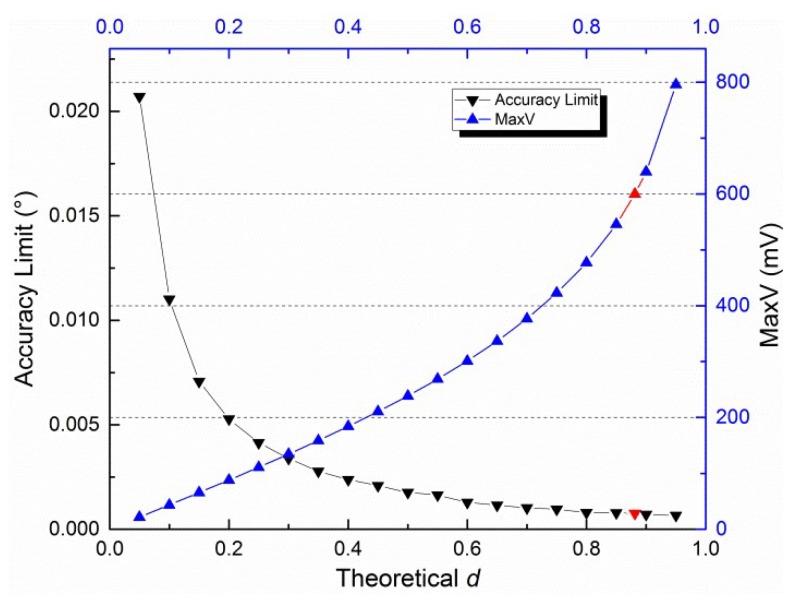
Maximum of *V_i_AD_* and accuracy limit for *θ* when *d* is between 0.05 and 0.95.

The maximum of *V_i_AD_* and the maximum absolute error (accuracy limit) of *θ*_12_ that change with *d* are shown in Figure 6. The accuracy limit for *θ*_13_ and *θ*_23_ is identical to that for *θ*_12_. Therefore, only one curve is shown. *V_ref_* is inserted (red) into Figure 6. The corresponding *d* for *V_ref_* is 0.8813. This number is high enough for outdoor measurement [31].

## 3. Calibration Method

### 3.1. Central-Symmetry Method with Integrating Sphere

In the calibration process, a precise rotary table is used to provide precise angle variations, which generate the accuracy of the polarization sensor. The centers of the integrating sphere (*O_IS_*), the polarization sensor (*O_S_*), and the rotary table (*O_R_*) should coincide, in ideal conditions. The central-symmetry method with the integrating sphere is proposed to improve calibration when the three centers do not coincide in the practical application.

**Figure 7:**
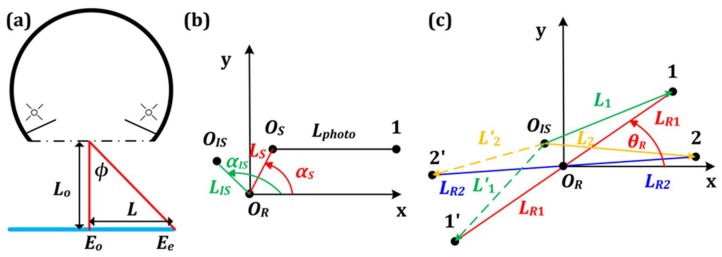
(**a**) Schematics illustrating the integrating sphere; (**b**) Schematics illustrating the eccentric feature of the three centers; (**c**) Schematics illustrating how to use the central-symmetry method in calibration.

Figure 7a shows a light-source diagram, where the black solid arc above the black dashed line represents the integrating sphere with lamps. The black dashed line represents the port of sphere. The blue solid line represents the projection of photosensitive surface of sensor. *L_o_* is the length between the port and the photosensitive surface. The irradiance at the center is *E_o_*. The irradiance at the off-axis edge is *E_e_*. *L* is the off-axis distance between the off-axis edge and the center. *Φ* is the intersection angle between the two lines in Figure 7a.

Figure 7b shows the eccentric features of the three centers on the photosensitive surface. *O_R_* is considered the center. *O_IS_* is at the position of *E_o_* in Figure 7a. *O_S_* is the center of circle, where the six photodetectors are located. The active photodetector area is considered as a point in the simulation. Point 1 represents the position of photodetector 1. *L_photo_* is 14 mm. The coordinate system *O_R_*-*XY* is built. Axis *X* is parallel to Line *O_S_*1. The eccentric distances of the integrating sphere and the sensor are *L_IS_* and *L_S_*, respectively. The eccentric angles are *α_IS_* and *α_S_*, respectively. This figure is also the initial state of the anticlockwise rotation in Section 3.2.

Figure 7c shows how the central-symmetry method is used in the rotation. Rotating the sensor by 180° around *O_R_*, Points 1 and 2 results in positions 1’ and 2’. The off-axis distances between Point 1, 2, 1’, and 2’ and *O_IS_* are *L*_1_, *L*_2_, *L*_1_’, and *L*_2_’, respectively. The eccentric distances of Points 1 (1’) and 2 (2’) are *L_R_*_1_ and *L_R_*_2_, respectively. Assuming that the scattered light in Figure 1b is the light from the sphere, the positions of the six photodiodes with respect to the sphere can be visualized with the aid of Figure 1b,c.

According to the integrating sphere manual, the irradiance (W/m^2^) at the off-axis edge can be expressed as *E_e_* = *E_o_*cos^4^*φ*. Then, the irradiances at Points 1, 2, 1’ and 2’ are:

(4) $$\begin{array}{l} E_{1}=E_{o}\cos^{4}\varphi_{1}\;=E_{o}{\left(L_{o}/\sqrt{L_{1}{}^{2}+L_{o}{}^{2}}\right)}^{4}\\ E_{2}=E_{o}\cos^{4}\varphi_{2}\;=E_{o}{\left(L_{o}/\sqrt{L_{2}{}^{2}+L_{o}{}^{2}}\right)}^{4}\\ {E^{\prime }}_{1}=E_{o}\cos^{4}{\varphi^{\prime }}_{1}\;=E_{o}{\left(L_{o}/\sqrt{{L^{\prime }}_{1}{}^{2}+L_{o}{}^{2}}\right)}^{4}\\ {E^{\prime }}_{2}=E_{o}\cos^{4}{\varphi^{\prime }}_{2}\;=E_{o}{\left(L_{o}/\sqrt{{L^{\prime }}_{2}{}^{2}+L_{o}{}^{2}}\right)}^{4} \end{array}.$$

The voltage derived from the amplifier of photodetectors 1 and 2, before rotation, is *V*_1*br*_, while the voltage after rotation is *V*_1a*r*_. Assuming that the spectral responsivity (A/W) and the active area size (m^2^) for each photodiode are the same, we obtain:

(5) $$\begin{array}{l} V_{1br}=V_{1\_ideal}+0.5\log(E_{1}/E_{2})\\ V_{1ar}=V_{1\_ideal}+0.5\log({E^{\prime }}_{1}/{E^{\prime }}_{2}) \end{array}.$$

The mean value (*V*_1*mr*_) of two voltages is used—and not *V*_1*br*_ or *V*_1a*r*_—for calibration using the central-symmetry method for indoor experiments.

### 3.2. Simulation of the Central-Symmetry Method

The performance of the central-symmetry method, namely the voltage deviation between *V_mr_* and *V_ideal_*, was studied in detail during the simulation. During the anticlockwise rotation in Figure 7c, the polarization angle, namely the rotational angle (*θ_R_*) of the precise rotary table, kept varying and had a big influence on the voltage deviation. However, the polarization degree (*d*) had no influence on the voltage deviation, according to Equation (5). Four parameters (*L_IS_*, *L_S_*, *α_IS_*, and *α_S_*) were investigated, and *L_o_* was set to 20 mm.

**Figure 8:**
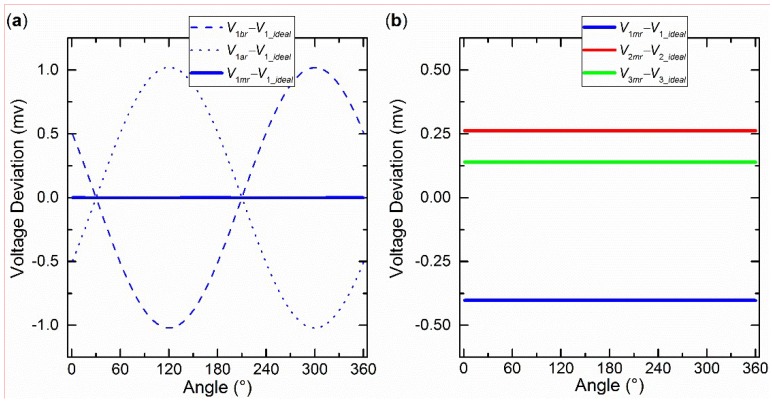
(**a**) The separate influence of *O_IS_* when *L_IS_* = 0.05 mm and *α_IS_* = 0°. (**b**) The separate influence of *O_S_* on voltages when *L_S_* = 0.02 mm and *α_S_* = 70°.

First, the influences of the two eccentric centers (*O_IS_* and *O_S_* in Figure 7b) on the voltage were analyzed separately. Figure 8a shows the separate influence of *O_IS_*, when *O_S_* and *O_R_* coincide. The voltage deviations between *V*_1*a*r_ and *V*_1_*ideal*_, and *V*_1*b*r_ and *V*_1_*ideal*_ can be considered approximate sine curves. It is easy to conclude that the amplitude depends on the eccentric distance (*L_IS_*) and the phase depends on the eccentric angle (*α_IS_*). The other two groups of voltage deviations were omitted because of the similarity. The voltage deviation between *V*_1*mr*_ and *V*_1_*ideal*_ was close to 0, which proved the validity of the central-symmetry method preliminarily. Figure 8b shows the separate influence of *O_S_*, when *O_IS_* and *O_R_* coincide. The three deviations between *V_mr_* and *V_ideal_* seemed constant. Under this condition (*L_IS_* = 0 mm), *V_ar_* was the same as *V_br_*, according to Equation (5)_._ Therefore, they were both the same as *V_mr_*. The other two groups of deviations were omitted.

**Figure 9:**
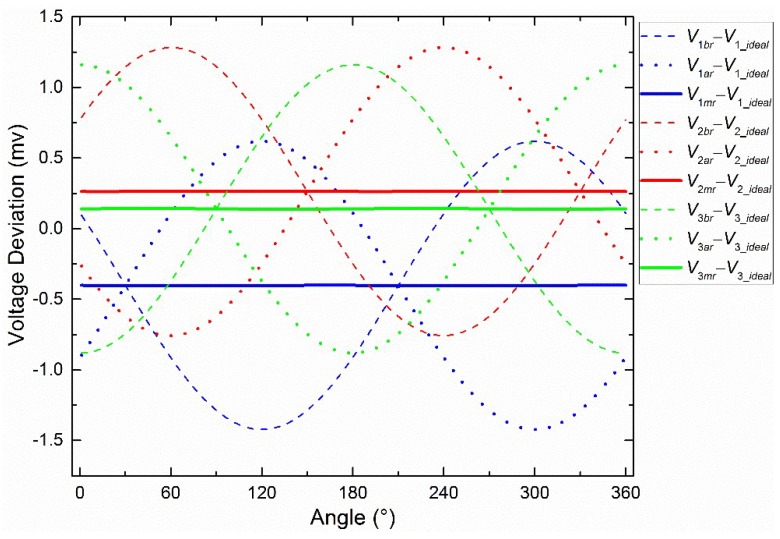
The integrated influence of *O_IS_* and *O_S_* when *L_IS_* = 0.05 mm, *α_IS_* = 0°, *L_S_* = 0.02 mm, and *α_S_* = 70°.

Second, the integrated influence of the two eccentric centers on the voltage is shown in Figure 9. It was found that Figure 9 was the superposition of Figure 8a,b. The influences of the two eccentric centers were not stuck to each other. On the contrary, they were independent of each other.

Third, the analytical expression was used to explain the geometric patterns in Figure 8 and Figure 9, and the general regular was proposed. The deviations between *V*_1*mr*_ and *V*_1_*ideal*_ in the four calculation steps are shown as Equation (6): three intermediate results and one final result. Substituting *V*_1*br*_ and *V*_1a*r*_ with Equations (4) and (5), the first intermediate result was obtained. During the anticlockwise rotation in Figure 7c, the off-axis distances (*L*_1_, *L*_2_, *L*’_1_, and *L*’_2_) can be calculated using the coordinates of the moving points (1 and 2) and the fixed points (*O_IS_*) directly, because the moving points can be calculated easily with the constant eccentric distances (*L_R_*_1_ and *L_R_*_2_). Therefore, the second intermediate result was obtained. An approximate assumption in Equation (7) was proposed to obtain the third intermediate result, because *L_IS_* was chosen to be 0.02 mm, *L_R2_* was 14 mm, and *L_o_* was 20 mm. Finally, the simplification of the deviation between *V*_1*mr*_ and *V*_1_*ideal,*_ based on the assumption, was found without *θ_R_* or *α_IS_*. Hence, the deviation was constant, as the solid lines shown in Figure 8a,b and Figure 9. In addition, it is 0 if *L_R_*_1_ is equal to *L_R_*_2_, as shown in Figure 8a.

(6) $$\begin{array}{c} V_{1mr}-V_{1\_ideal}=0.5\log\frac{\left({L_{2}}^{2}+{L_{0}}^{2}\right)\left({{L^{\prime }}_{2}}^{2}+{L_{0}}^{2}\right)}{\left({L_{1}}^{2}+{L_{0}}^{2}\right)\left({{L^{\prime }}_{1}}^{2}+{L_{0}}^{2}\right)}\\ =0.5\log\frac{({L_{0}}^{2}+{L^{2}}_{IS}+{L^{2}}_{R2}{}^{2}-4{L^{2}}_{R2}{L^{2}}_{IS}\cos^{2}\left(\theta_{R}-60-\alpha_{IS}\right)}{({L_{0}}^{2}+{L^{2}}_{IS}+{L^{2}}_{R1}{}^{2}-4{L^{2}}_{R1}{L^{2}}_{IS}\cos^{2}\left(\theta_{R}-\alpha_{IS}\right)}\\ \approx 0.5\log\frac{({L_{0}}^{2}+{L^{2}}_{IS}+{L^{2}}_{R2}{}^{2}}{({L_{0}}^{2}+{L^{2}}_{IS}+{L^{2}}_{R1}{}^{2}}\;\;\\ =\log\frac{{L_{0}}^{2}+{L^{2}}_{IS}+{L^{2}}_{R2}}{{L_{0}}^{2}+{L^{2}}_{IS}+{L^{2}}_{R1}} \end{array},$$

(7) $$\begin{array}{l} \mathrm{If}\;L^{2}{}_{IS}\ll L_{0}{}^{2}+L^{2}{}_{IS}+L^{2}{}_{R2}\\ \mathrm{Then}:4L^{2}{}_{R2}L^{2}{}_{IS}\cos^{2}(\theta_{R}-60-\alpha_{IS})\approx 0\\ \;4L^{2}{}_{R1}L^{2}{}_{IS}\cos^{2}(\theta_{R}-\alpha_{IS})\approx 0. \end{array}$$

Based on Equation (6), there was a general regular: The method optimized the nonconstant voltage deviations generated by the off-axis distance of the integrated sphere during rotation. Constant voltage deviations, which can be calibrated easily, were the final outputs of the central-symmetry method during rotation.

In addition, *L_R_*_1_ and *L_R_*_2_ in Equation (6) were expanded further. Then we obtained:

(8) $$V_{1mr}-V_{1\_ideal}=\log\frac{L_{0}{}^{2}+L^{2}{}_{IS}+L^{2}{}_{S}+L^{2}{}_{photo}+2L_{S}\cos(\alpha_{S}+60)}{L_{0}{}^{2}+L^{2}{}_{IS}+L^{2}{}_{S}+L^{2}{}_{photo}+2L_{S}\cos\alpha_{S}}.$$

**Figure 10:**
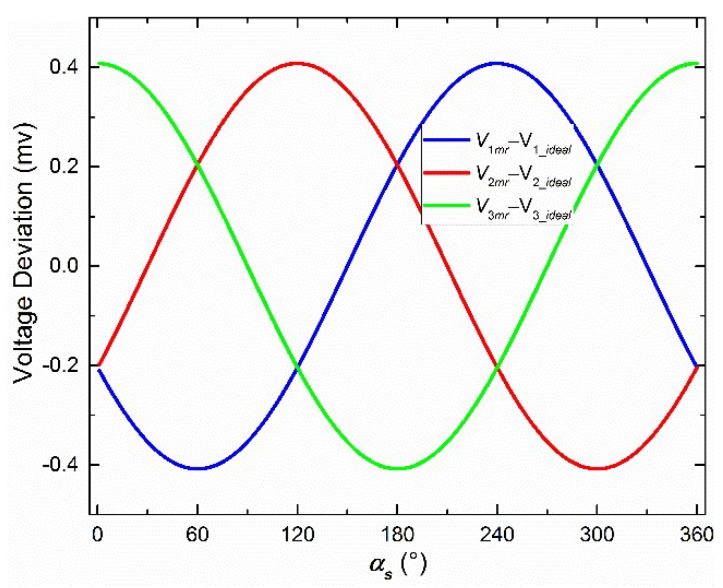
The influence of *α_S_* on the three voltage deviations after using the central-symmetry method.

A new assumption, as shown in Equation (9), was proposed to investigate the derivation of Equation (8) when *α_S_* is the unknown parameter. As a result, when *α_S_* was 240°, Equation (8) reached the maximum. The three deviations between *V_mr_* and *V_ideal_* are as shown in Figure 10 when the resolution of *α_S_* is 1°.

(9) $$\mathrm{If}\;2L_{S}<<L^{2}{}_{IS}+L^{2}{}_{S}+L^{2}{}_{photo}+L_{0}{}^{2}\;\mathrm{Then}:\frac{2L_{S}}{L^{2}{}_{IS}+L^{2}{}_{S}+L^{2}{}_{photo}+L_{0}{}^{2}}\approx 0$$

**Figure 11:**
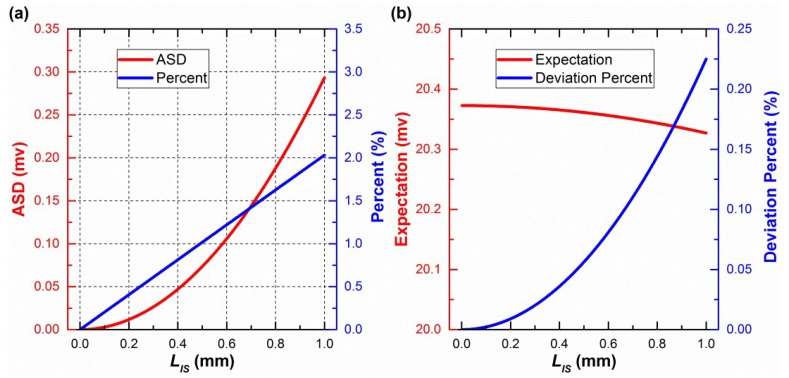
After using the central-symmetry method: (**a**) The influence of *L_IS_* on ASD and percent; (**b**) the influence of *L_IS_* on expectation and deviation percent.

**Figure 12:**
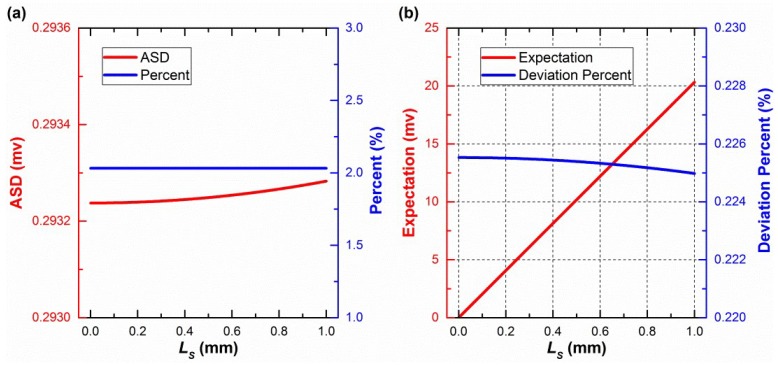
After using the central-symmetry method: (**a**) The influence of *L_S_* on ASD and percent; (**b**) the influence of *L_S_* on expectation and deviation percent.

Fourth, the standard deviations of voltage deviations were used to evaluate the central-symmetry method, which does not change mathematical expectations. During a rotation of 360° with 1° intervals, the number of samples is 360. Standard deviations were investigated when *L_S_* and *L_IS_* were both between 0.001 and 1 mm. The standard deviations, before and after the central-symmetry method is used, are defined as BSD and ASD, respectively. ASD, the percent of ASD to BSD, the expectation, and the deviation percent of the expectation are shown in Figure 11, when *L_S_* is 1 mm and *L_IS_* is between 0.001 and 1 mm, and in Figure 12 when *L_S_* is between 0.001 and 1 mm and *L_IS_* is 1 mm, respectively. Only the first one of the three voltage deviations was chosen to be studied because of the similarity between them. *α_S_* was set to be 240° because the corresponding voltage deviation was the maximum, as shown in Figure 10.

In Figure 12a, ASD and the percent vary very slightly and can be approximately considered as a constant value. This also happens when *L_IS_* is another value between 0.001 and 1 mm. The smaller the *L_IS_*, the more precise is this phenomenon. Therefore, it is concluded that *L_S_* hardly makes an effect on ASD and the deviation percent when *L_S_* and *L_IS_* are between 0.001 and 1 mm. Therefore, as shown in Figure 11a, the standard deviations have been largely reduced by the central-symmetry method in the simulation. This result supports the general regular derived from Equation (6).

A similar conclusion to the ASD is made that *L_IS_* hardly affects expectations, based on the red curve in Figure 11b. Therefore, Figure 12b shows the influence of *L_S_* on the expectations of voltage deviation when *L_S_* and *L_IS_* are between 0.001 and 1 mm. The expectations will be handled in the following calibration part.

The expectations remain unchanged when the method is used. During the 360° rotation, the expectations of *V_br_* and *V*_a*r*_ are the same, because *V_br_* and *V*_a*r*_ contain the same elements, which are exactly one period (360°) of a periodic function. The difference is that there is a 180° phase deviation. Then, the expectations of *V_br_*–*V_ideal_* and *V*_a*r*_–*V_ideal_* are the same. Therefore, the expectations of *V_mr_*–*V_ideal_* are the same as *V_br_*–*V_ideal_* or *V*_a*r*_–*V_ideal_*. In other words, the central-symmetry method does not change the expectations of voltage deviation.

To further verify Equation (6), the “constant” values calculated using Equation (6) are added to both Figure 11b and Figure 12b. To be exact, the deviation percent between the “constant” values (*CV*) and the expectations (*ME*), namely (*CV*–*ME*)/*ME* × 100%, was investigated. The deviation percentages are below 0.23% in Figure 11b and Figure 12b, which verify Equation (6).

At the beginning of this part, *L_o_* was set to 20 mm, which is close to the actual value. If its value meets the requirements of Equation (7), the above conclusion of the method can still be made. Because *L_R_*_2_ itself has already met the requirements of Equation (7), *L_o_* is allowed to be any real number. We suggest that it should be [0, 40] mm.

### 3.3. Noncontinuous Method

**Figure 13:**
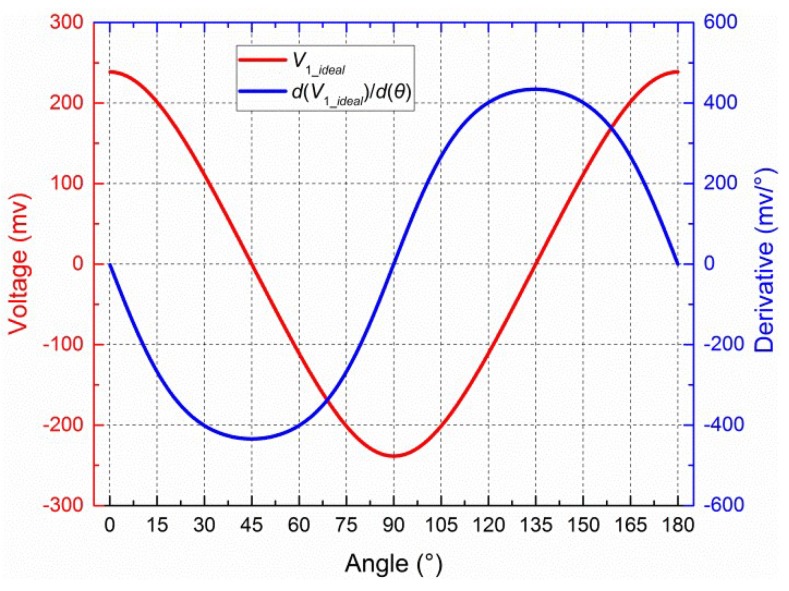
*V*_1_*ideal*_ and its derivative when *d* = 0.5.

Not all voltages determined using the central-symmetry method were used in calibration parameter estimation. More precisely, voltages with small derivatives (d*V*/d*θ*) were rejected, and only the remaining values were used to calculate the calibration parameter. The derivative is given by Equation (10). The voltage (*V*_1_*ideal*_) and its derivative are shown in Figure 13 when *d* is 0.5. Generally, when voltages are close to 0, the corresponding derivatives are close to their extreme values. When voltages are close to their extreme values, the corresponding derivatives are close to 0. The mathematical regular between voltages and their derivatives is similar to a sine function. Note that at least six points remained; otherwise, a poor result will be obtained. More details are shown in Section 4.4.

(10) $$\frac{dV_{1\_ideal}}{d\theta }=\frac{2d\sin2\theta }{\ln10\times (d^{2}\cos^{2}2\theta -1)}.$$

### 3.4. Calibration Parameters

The incident skylight of the polarization sensor includes two components: unpolarized light and totally polarized light. The two respective gains [32] can be described as follows:

(11) $$\begin{array}{l} g_{up}=(\tau_{M}+\tau_{m})/2=\bar{\tau }\\ g_{tp}=\tau_{M}\cos^{2}\theta +\tau_{m}\sin^{2}\theta \end{array},$$

where *g_up_* represents the gain of unpolarized light; *g_tp_* is the gain of totally polarized light. *τ_M_* is the transmittance when the reference angle and main polarization angle of incident light are parallel. *τ_m_* is the transmittance when the two directions are orthogonal.

Then, the irradiance at one photodiode and the output of one photodiode are as follows:

(12) $$E_{p}=\tau_{f}\tau_{c}E_{in}((1-d)\bar{\tau }+d(\tau_{M}\cos^{2}\theta +\tau_{m}\sin^{2}\theta )),$$

(13) $$I_{out}=s_{r}A_{r}E_{p},$$

where *E_in_* is the irradiance of incident light. *τ_f_* is the transmittance of the corresponding blue filter. *τ_c_* is the transmittance of the corresponding plano-convex lens. *s_r_* is the spectral responsivity of photodiode. *A_r_* is the active area size of photodiode. Then, *V_i_* can be formulated as follows:

(14) $$\begin{array}{l} V_{i}=0.5k_{vi}\log k_{\tau i}\frac{1+d(1+k_{dj})\cos(2\theta +\alpha_{j})}{1-d(1+k_{dh})\cos(2\theta +\alpha_{h})}+k_{ci}\\ k_{\tau i}=\frac{0.5\tau_{fj}\tau_{cj}s_{rj}A_{rj}E_{ij}(\tau_{Mj}+\tau_{mj})}{0.5\tau_{fh}\tau_{ch}s_{rh}A_{rh}E_{ih}(\tau_{Mh}+\tau_{mh})}\\ k_{dj}=-2\tau_{mj}/(\tau_{Mj}+\tau_{mj}) \end{array},$$

where *j* is 2*i* – 1; *h* is equal to 2*i*. *k_v_*_1_ is the deviation parameter of reference voltage of logarithmic amplifier. The installation angles of polarizer 0° and polarizer 90° are *α*_1_ and *α*_2_, respectively. *k_ci_* is the constant value generated by the integrating sphere method, namely the expectation in Figure 12b. *k_di_* is the coefficient of the non-ideal polarizer.

Then, Equation (1) can be written as follows:

(15) $$V_{i}=0.5k_{vi}\log\frac{1+d(1+k_{dj})\cos(2\theta +\alpha_{j})}{1-d(1+k_{dh})\cos(2\theta +\alpha_{h})}+k_{i}.$$

Additionally, *k_i_* is:

(16) $$k_{i}=0.5k_{vi}\log k_{\tau i}+k_{ci}.$$

To obtain the final output *θ*, Equation (15) should be transformed as follows.

(17) $$A_{i}=\frac{\left(1+d(1+k_{dj})\cos(2\theta +\alpha_{j}\right)}{\left(1-d(1+k_{dh})\cos(2\theta +\alpha_{h}\right)}=10^{2(V_{i}-k_{i})/k_{vi}}.$$

By eliminating the unknown number *d* and expanding all cosine functions, we obtain:

(18) $$\begin{array}{l} \theta_{i(i+1)}=\frac{1}{2}\arctan\frac{\left(A_{i}-1)(A_{i+1}wc_{h+2}+wc_{j+2})-(A_{i+1}-1)(A_{i}wc_{h}+wc_{j}\right)}{\left(A_{i}-1)(A_{i+1}ws_{h+2}+ws_{j+2})-(A_{i+1}-1)(A_{i}ws_{h}+ws_{j}\right)}\\ wc_{m}=(1+k_{dm})\cos\alpha_{m}\\ ws_{m}=(1+k_{dm})\sin\alpha_{m}\;m\in [1,2,3,4,5,6] \end{array}.$$

### 3.5. Decoupling Method for Calibration Parameters

The iterative least-squares estimation method was used to obtain the calibration parameters. There are four types of calibration parameters (*k*, *k_v_*, *k_d_*, *α*) (see Equation (15)). Among them, *k*, *k_v_*, and *α* are easy to obtain. However, the exact value for *k_d_* cannot be determined. Clearly, *d* and (1 + *k_d_*) are coupled, because there are seven unknown numbers (*d* and *k_d_*_1-6_) but only six equations (see Equation (19)). Therefore, the decoupling method is necessary. The coupled parts are as follows:

(19) $$d(1+k_{dm})=d_{cm},$$

where *d_c__m_* is obtained using the iterative least-squares estimation method. The value (*d_a_*) for *d* was determined by the authors and is not an exact value obtained by an algorithm of decoupling method. Note that *d_a_* cannot be zero. Then, we can obtain *k_d_*.

(20) $$k_{dm}=\frac{d_{cm}}{d_{a}}-1.$$

Then, Equation (15) becomes:

(21) $$V_{i}=0.5k_{vi}\log\frac{1+d_{cj}\cos(2\theta +\alpha_{j})(d/d_{a})}{1-d_{ch}\cos(2\theta +\alpha_{h})(d/d_{a})}+k_{i}.$$

The unknown variable in Equation (21) can be considered as *θ* and *d*/*d_a_*. The inaccurate *d_a_* is a part of the unknown variables and does not affect other calibration parameters. Therefore, it can be concluded that the decoupling method does not affect *θ* but *d*.

## 4. Indoor Results

**Figure 14:**
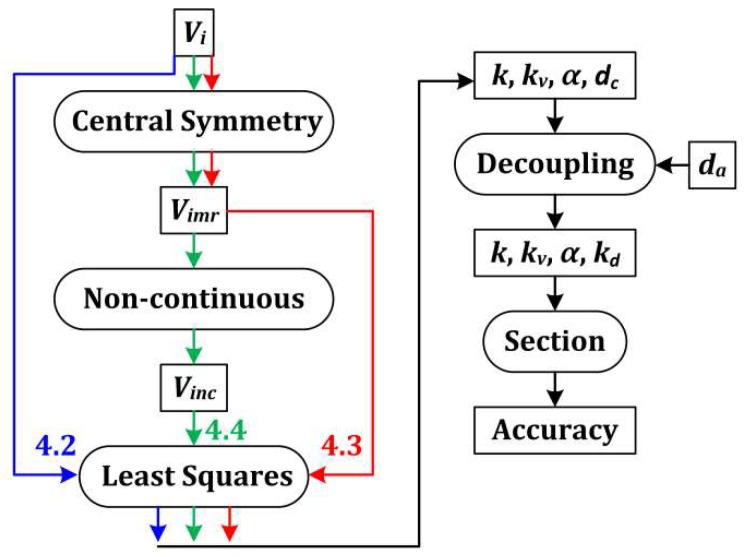
Overview of three types of calibrations.

The indoor calibration experiments were performed and the performances of different methods in Section 3 were analyzed by three different types of calibrations, as shown in Figure 14. Before the least squares were used to calculate the calibration parameters, the three calibrations were distinguished by arrows with different colors. After the least squares, they shared the same process.

The first type of calibration, which is indicated in Section 4.2 and is shown by the blue arrows and the black arrows, only included the section algorithm. The voltages were sent to the least squares directly. The same process shown by the black arrows was a combination of Section 2.3, Section 3.4, Section 3.5 and Section 4.5. Section 4.5 indicates “Accuracy”. Section 2.3 indicates “Section”. Section 3.4 and Section 3.5 indicate the remaining part.

The central symmetry method was inserted into the second type of calibration in Section 4.3, shown by the red arrows. The central symmetry method and the noncontinuous method were both inserted into the third type of calibration in Section 4.4, shown by the green arrows.

### 4.1. Calibration Device

**Figure 15:**
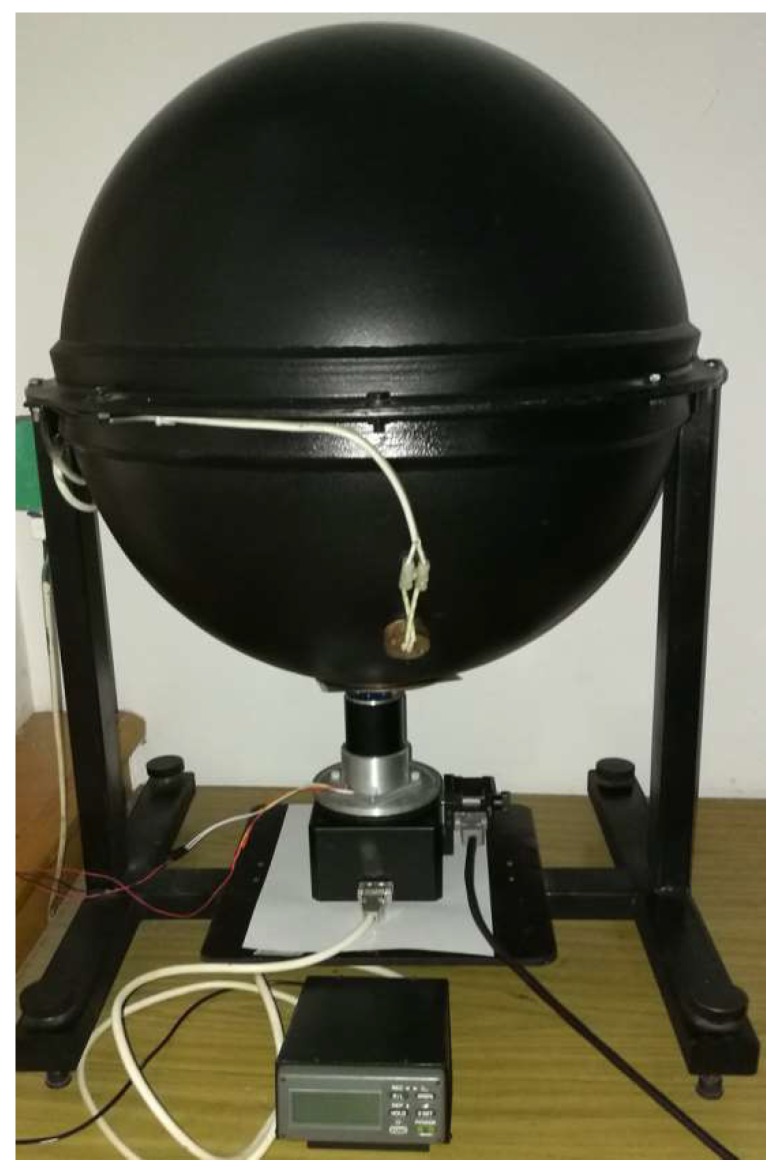
Photograph of indoor calibration setup.

The calibration tools, as shown in Figure 15, include an integrating sphere and a precise rotary table. The integrating sphere provides uniform light, which is unpolarized. A linear polarizer is fixed at the port of the sphere. Then, the light from the sphere becomes linear polarization light after passing through the polarizer. Note that the lamps in the sphere need at least 15 min before using, otherwise the light intensity might not be stable. The model of the sphere is JF-500 (Hefei Xingyue Luminous Technology Applications Institute, Hefei, China). Its diameter is 500 mm. The diameter of its port, where light comes out, is 100 mm. The rotary table, equipped with an optical incremental encoder, has a 1″ accuracy (0.000277°).

The rotary table stops for 1 s every 10° with an angular speed of 5°/s, and a full range of 360°. As a result, one experiment lasts for 108 s. The values derived from the encoder are considered as “theoretical values”. The mean values of 40 samples at each position were recorded as the measured values.

### 4.2. Section-Only Algorithm

**Figure 16:**
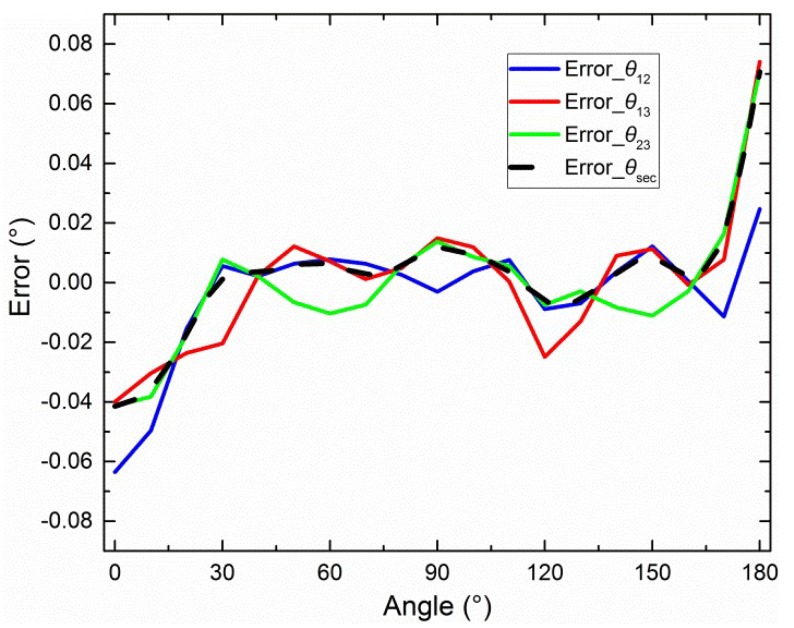
Error curves when only the section algorithm is used.

The error curves for *θ*_12_, *θ*_13_, *θ*_23_, and *θ*_sec_ after calibration, without using the integrating sphere central-symmetry method but by using the section algorithm, are shown in Figure 16.

### 4.3. Adding the (Integrating-Sphere) Central-Symmetry Method

**Figure 17:**
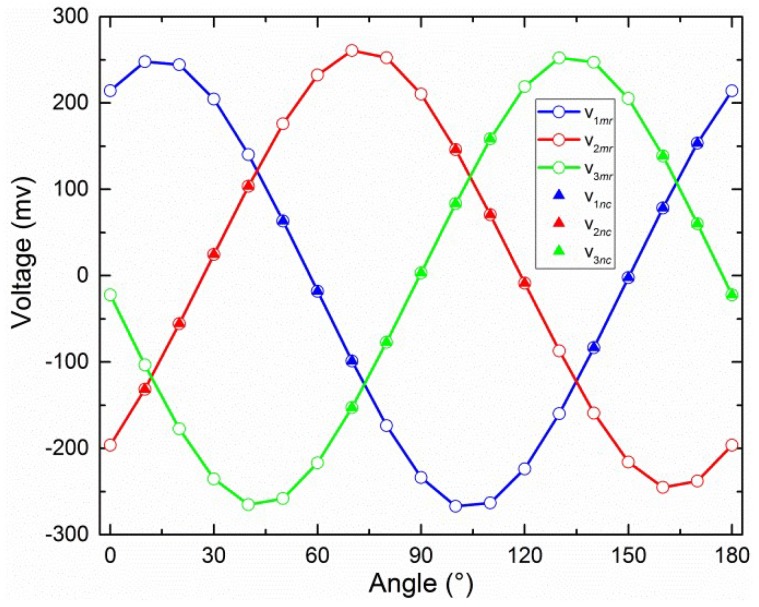
Estimated voltage curves for Section 4.3 and noncontinuous points for Section 4.4.

The 57 (3 × 19) points (*V_mr_*) were obtained using the central-symmetry method (see Figure 17).

**Figure 18:**
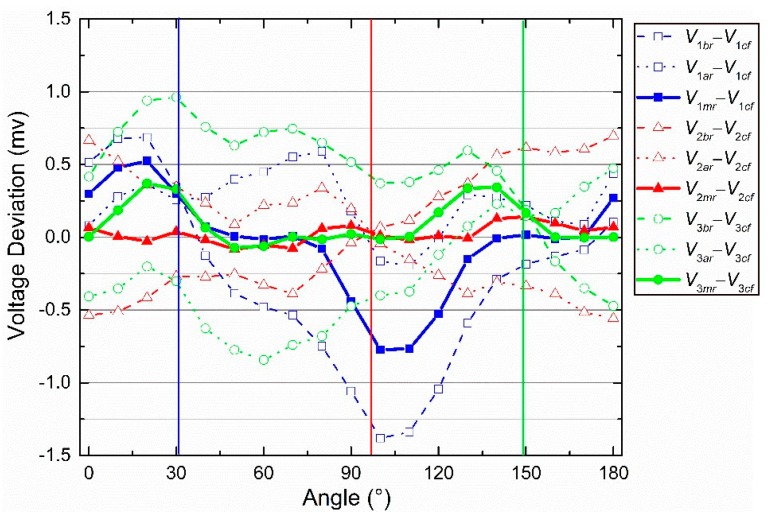
Experimental data analysis when the central-symmetry method is also used.

**Figure 19:**
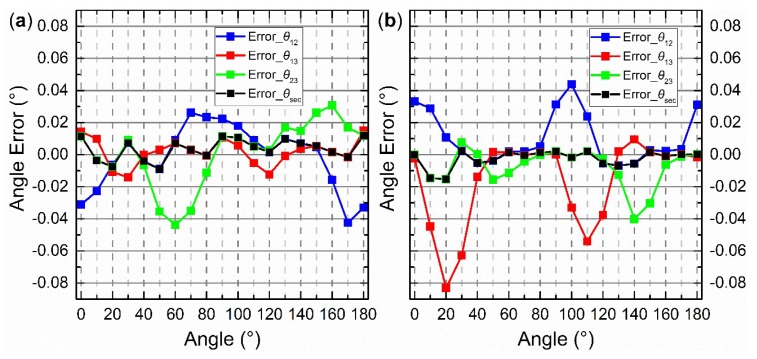
(**a**) Error curves when the central-symmetry method is also used; (**b**) error curves when the noncontinuous method is added.

The performance of the central-symmetry method is shown in Figure 18. V*_icf_* are the outputs of Equation (21) when the inputs are the angle provided by the precise rotary table. The calibration parameters in Equation (21) are the indoor parameters as shown in Table 2. V*_icf_* are considered as ideal values, corresponding to V*_i_ideal_* in Figure 9, in this performance analysis. The three vertical lines with red, blue, and green color are used to indicate three intersection points (see more details in the discussion). The error curves for *θ*_12_, *θ*_13_, *θ*_23_, and *θ*_sec_, after calibration, using both the central-symmetry method and section algorithm are shown in Figure 19a.

### 4.4. Adding the Noncontinuous Method

The points (*V_nc_*), marked as triangles, were used with the iterative least-squares estimation to obtain the calibration parameters (see Figure 17). The error curves for *θ*_12_, *θ*_13_, *θ*_23_, and *θ*_sec_ after calibration, obtained using the central-symmetry method, noncontinuous method, and section algorithm are shown in Figure 19b. Note that the start point of the *x*-axis was normalized from −10.3803° to 0°. The 19 origin actual theoretical polarization angles are −10.3803°, −0.3781°, 9.6292°, 19.6247°, 29.6280°, 39.6358°, 49.6294°, 59.6338°, 69.6377°, 79.6341°, 89.6369°, 99.6438°, 109.6430°, 119.6375°, 129.6430°, 139.6358°, 149.6355°, 159.6300°, 169.6194° in Figure 16, Figure 17, Figure 18 and Figure 19. According to the actual angles, how the section algorithm works can be seen.

### 4.5. Comparison of Three Calibration Methods

**Figure 20:**
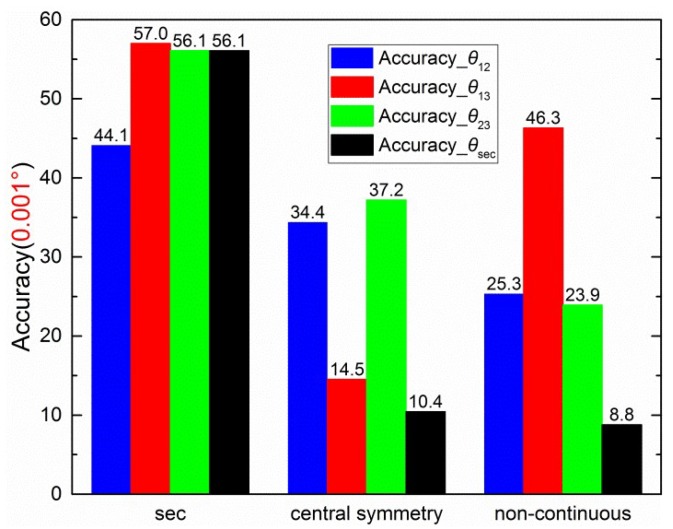
Summary of accuracies of three types of calibration.

To compare the calibration results, the half value of difference between the maximum and minimum in the error curve is defined as the accuracy of the sensor. For example, the maximum and minimum of black dashed error curve (Error_*θ*_sec_) in Figure 19 are 0.00226° and −0.01530°, respectively. Then, the calibration accuracy is ±0.0088°. The calibration results of Section 4.2, Section 4.3 and Section 4.4 are summarized in Figure 20.

### 4.6. Analysis of Four Calibration Parameters

The calibration parameters derived from Section 4.4 are considered as the indoor calibration parameters because of the highest accuracy. They are shown in the third line in Table 2. The theoretical calibration parameters are shown in the second line.

**Figure 21:**
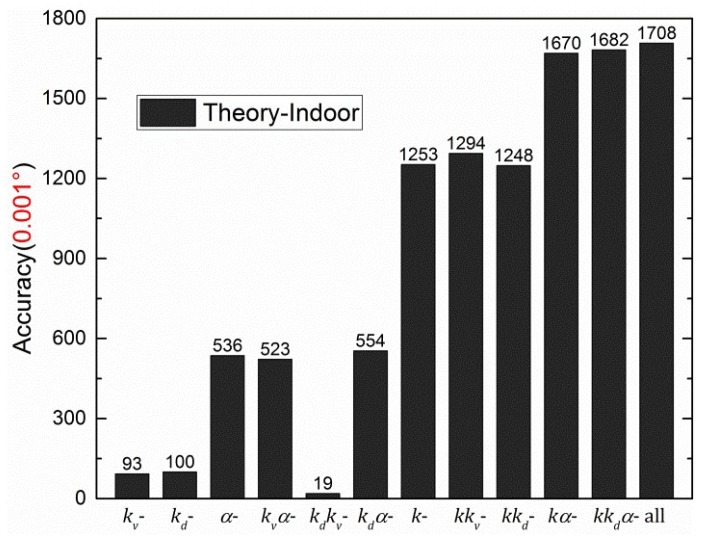
Theoretical calibration parameters replacing the corresponding indoor calibration parameters.

Figure 21 shows the accuracies, when parts of the indoor calibration parameters shown in Table 2 were replaced with the corresponding theoretical calibration parameters. For example, the fifth column “*k_d_k_v_*-” represents the accuracy when indoor *k_d_* and *k_v_* were replaced with the corresponding theoretical calibration parameters (0 and 1), respectively. Furthermore, “-” was used to distinguish the comments between different columns. Considering the accuracy limit in Figure 6, all accuracies are correct to three decimal places when the unit is 1°. For example, the original accuracy of the indoor calibration was ±0.0088°, and the accuracy limit was about ±0.001° when *d* was ~0.7. As a result, the accuracy was ±0.009° (see the last row in Table 2). The unit for accuracy is 0.001° in Figure 21.

## 5. Outdoor Results

### 5.1. Static Outdoor Experiments

**Figure 22:**
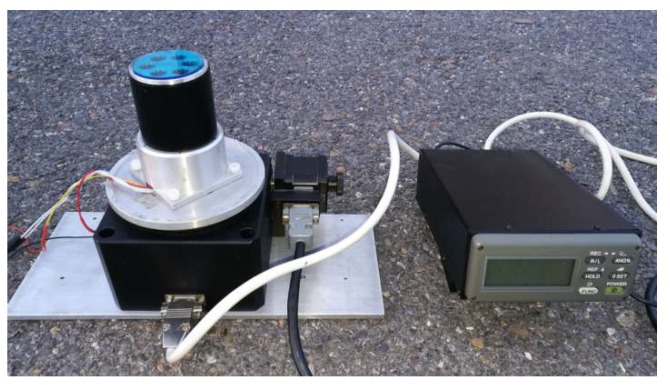
Photograph of outdoor setup.

**Figure 23:**
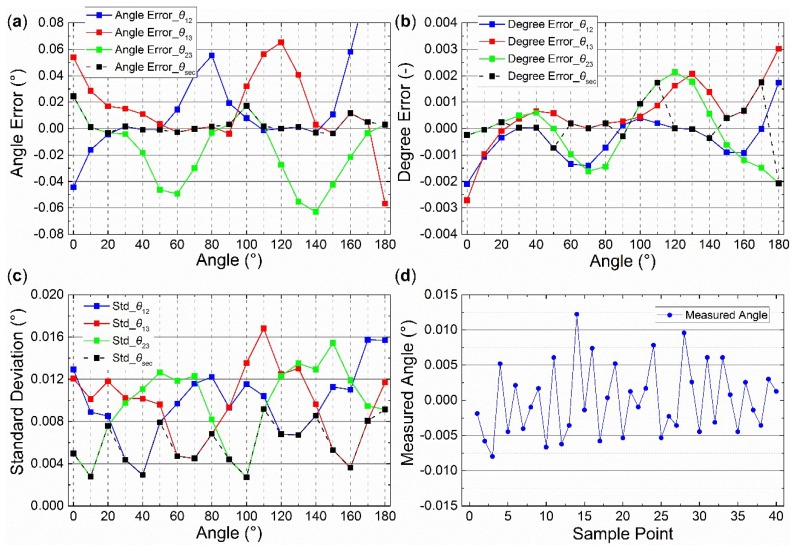
Outdoor2 errors after calibration at 16:43:08. (**a**) Angle error; (**b**) degree error; (**c**) standard deviation; (**d**) the 40 measured samples.

Two groups of static outdoor experiments were performed on the roof (121°32’38’’E, 38°52’45’’N) of the School of Mechanical Engineering at Dalian University of Technology on November 16, 2018 (see Figure 22). The sky was clear and the polarization degree was high (from 0.65 to 0.71). The rotary table followed the same process as the indoor experiments. The end time of the first group was 16:11:28 (Beijing time). The end time of the second group was 16:43:08 (Beijing time). Each experiment lasted 108 s for a 360° range, and the data were calibrated. Note that both the noncontinuous method and the section algorithm were used in the calibration. However, the central-symmetry method was not used because the light source was the light originating from the sky and not from the integrating sphere. The outdoor calibration parameters are shown in the fourth (outdoor1) and fifth columns (outdoor2) of Table 2. The error curves for outdoor2 experiment after calibration are shown in Figure 23. The polarization angle error, the polarization degree, and the standard deviation of 40 samples are shown in Figure 23a–c, respectively. The 40 samples at the start point in Figure 23a–c are shown in Figure 23d. The degree error was ±0.003° when outdoor2 *d*_a_ (0.71) in Table 2 was considered the theoretical value. Most standard deviations were smaller than 0.01°. The error curves for outdoor1 were omitted, because they were very similar. The polarization angle of sky (the reference in this calibration) was assumed to be constant during a short time, such as 54 s for a 180° range, to ensure that the way to calculate the accuracy is the same as the manner in [7,9,10,12].

Moreover, the outdoor calibration parameters, when the slight variation of the reference was compensated, are shown in the sixth (sun1) and the seventh column (sun2) in Table 2. According to the single-scattering Rayleigh model, the variation of the reference equals the variation of the solar azimuth when the sensor is measuring the zenith.

Note that, similar to Figure 19, the start point of the *x*-axis was normalized to 0°. The 19 origin actual theoretical polarization angles are 82.2772°, 92.2855°, 102.2847°, 112.2788°, 122.2894°, 132.2861°, 142.2780°, 152.2813°, 162.2777°, 172.2850°, 2.2763°, 12.2780°, 22.2711°, 32.2619°, 42.2766°, 52.2694°, 62.2608°, 72.2697°, 82.2633° in Figure 23a–c.

**Figure 24:**
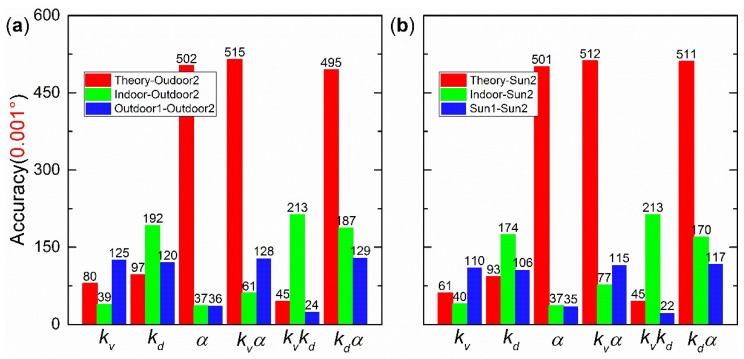
Outdoor replacement results without replacing *k*: (**a**) The compensation is not done; (**b**) the compensation is done.

**Figure 25:**
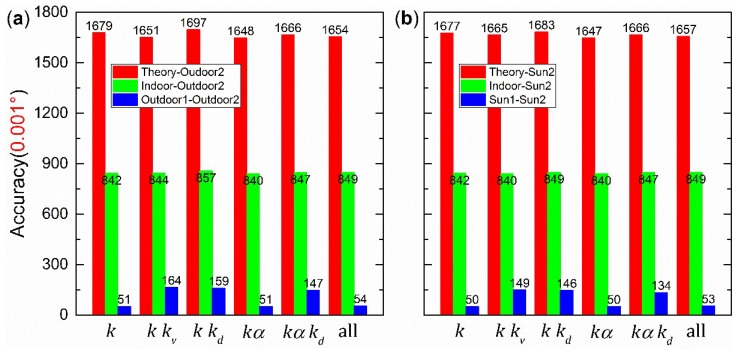
Outdoor replacement results when *k* was replaced: (**a**) The compensation is not done; (**b**) the compensation is done.

The same replacement method as in Section 4.6 was used to analyze the four calibration parameters. For example, the fifth column “*k_v_k_d_*” of Figure 24a is the accuracy, when outdoor2 *k_d_* and *k_v_* were replaced with the corresponding theoretical values (0 and 1), indoor or outdoor1 calibration parameters, respectively. The other parameters (*α* and *k*) were still the same for outdoor2. The results are divided into two parts, as shown in Figure 24a and Figure 25a, with respect to the calibration parameter *k* (i.e., *k_i_*), because the accuracy was lower when *k* was replaced. For all the replacements, all the measured data were the same as those in the experiment at 16:43:08. In Figure 24a and Figure 25a, the red, green, and blue columns represent that outdoor2 calibration parameters were replaced with the theoretical values, indoor, and outdoor1 calibration parameters, respectively. In Figure 24b and Figure 25b, the red, green, and blue columns represent that sun2 calibration parameters were replaced with the theoretical values, indoor, and sun1 calibration parameters, respectively.

### 5.2. Dynamic Outdoor Experiments

**Figure 26:**
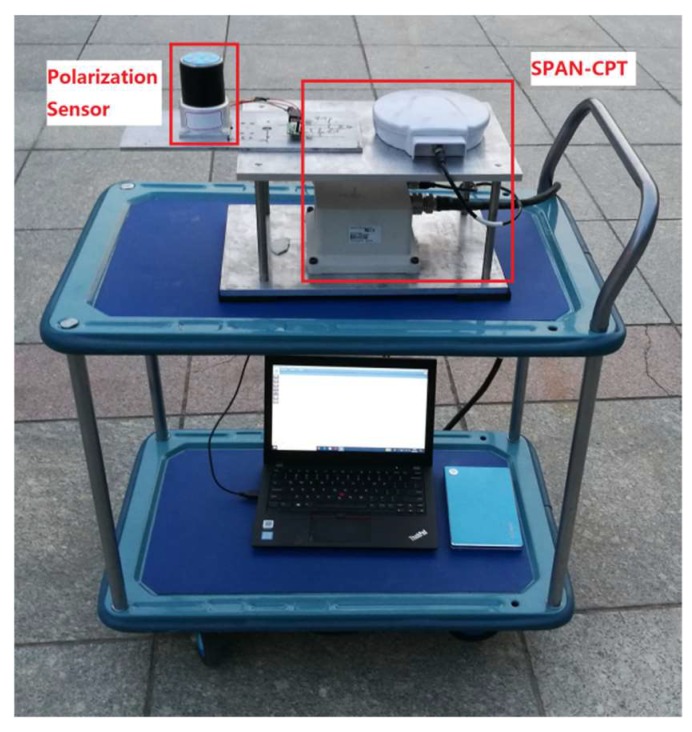
Photograph of dynamic outdoor setup.

**Figure 27:**
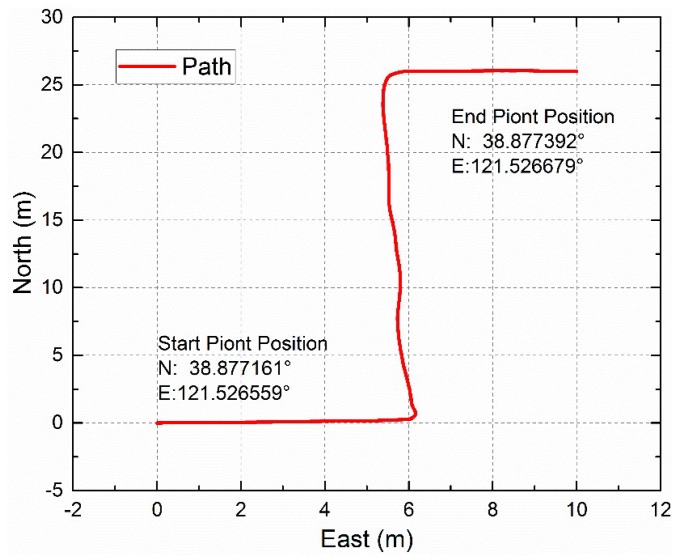
Outdoor path offered by the inertial navigation system.

**Figure 28:**
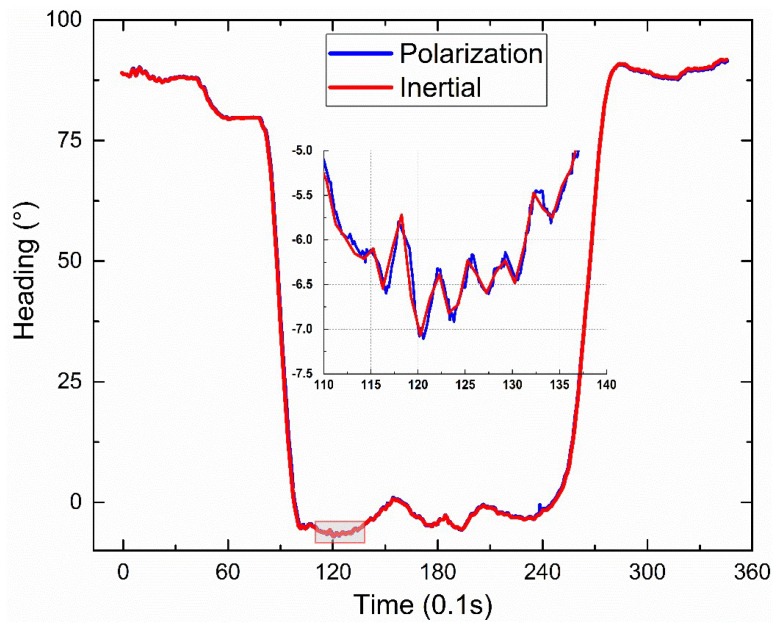
Headings of the polarization sensor and the inertial navigation system.

**Figure 29:**
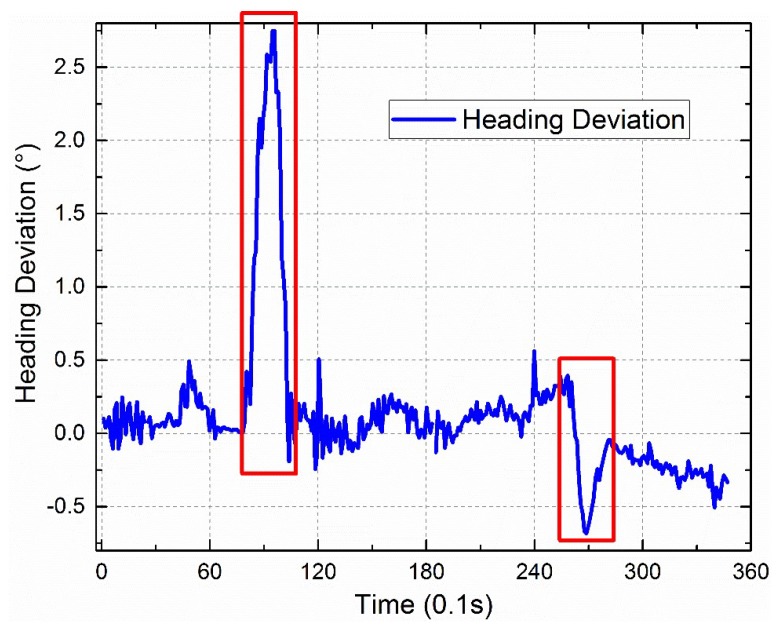
Heading deviations between the polarization sensor and the inertial navigation system.

The dynamic outdoor experiment was performed on the small square roof nearby the stadium of Dalian University of Technology at 19:01 on 1 July 2019 (Beijing time) (see Figure 26). The sky was clear and the polarization degree was high. The polarization sensor and the inertial navigation system with a high accuracy were fixed on the shelf, which has two layers. The shelf was fixed on the wheel barrow, which also has two layers. The path of the dynamic outdoor experiment was a “Z” path. Figure 27 shows the path offered by the inertial navigation system. Figure 28 shows both headings of the polarization sensor and the inertial navigation system, and the partial data is enlarged to show more details. Figure 29 shows their deviations. The polarization degree was about 0.61 during the dynamic outdoor experiment.

The inertial navigation system is SPAN-CPT, the price of which is 250,000 RMB or $36,700. SPAN is synchronized position attitude navigation and CPT is a specific type of SPAN. SPAN-CPT brings together two different but complementary positioning and navigation systems: Global Navigation Satellite System (GNSS) and an inertial navigation system (INS). By combining the best aspects of GNSS and INS into one system, SPAN technology offers a solution that is more accurate and reliable than either GNSS or INS alone. The heading accuracy can be ±0.06° if a good initial calibration is finished. The *Y*-axis points forward through the front of the vehicle, in the direction the vehicle is moving.

The way to obtain the head using the polarization sensor is the single-scattering Rayleigh model. The e-vector in the body coordinate system and the solar vector in the navigation coordinate system or the horizontal coordinate system are:

(22) $$\begin{array}{l} E_{b}=(\cos\theta ,\sin\theta ,0){\;}^{T}\\ S_{n}=(\cosh s\cos As,\cosh s\sin As,\cos hs){\;}^{T} \end{array},$$

where *As* is the solar azimuth and *hs* is the solar elevating angle. They can be calculated when the geographic position (latitude and longitude) and the time are known.

The solar vector in the body coordinate system is:

(23) $$S_{b}=C_{n}^{b}S_{n}.$$

The navigation coordinate system of SPAN-CPT has the definition: *Z*-axis pointing up; *Y*-axis pointing north; *X*-axis pointing east. Therefore, we can obtain:

(24) $$C_{n}^{b}=\left[\begin{array}{ccc} \cos\gamma & 0 & -\sin\gamma \\ 0 & 1 & 0\\ \sin\gamma & 0 & \cos\gamma \end{array}\right]\left[\begin{array}{ccc} 1 & 0 & 0\\ 0 & \cos\lambda & \sin\lambda \\ 0 & -\sin\lambda & \cos\lambda \end{array}\right]\left[\begin{array}{ccc} \cos H & -\sin H & 0\\ \sin H & \cos H & 0\\ 0 & 0 & 1 \end{array}\right],$$

where *γ* is the roll angle. *λ* is the pitch angle. *H* is the heading. Both the roll angle and the pitch angle are offered by SPAN-CPT.

Based on the single-scattering Rayleigh model, the e-vector is perpendicular to the solar vector. Then, we can obtain:

(25) $$E_{b}\cdot S_{b}=0.$$

The heading (*H*) can be calculated by Equations (22)–(25) because there is only one unknown number.

## 6. Discussion

The integer effect caused by the 16-bit ADC was considered in Figure 4 and Figure 5 in our simulation derived from Equation (3). Note that the max error is ±0.02° when *d* is equal to 0.05. In practice, electrical noise can strongly reduce the accuracy because the max *V* is very small (21.73 mV). In other words, the signal-to-noise ratio decreases. Methods to maintain the accuracy as high as when *d* is large (e.g., 0.5) when *d* is small will be investigated in a future study, where we will focus on improving the electronic circuit.

Figure 18 shows the deviations between *V_im_*_r_ and *V_icf_*, *V_ia_*_r_ and *V_icf_*, *V_ib_*_r_ and *V_icf_.* Overall, the experimental results are similar to the simulation results. In particular, the three red curves support the simulation in Figure 9 well. Figure 9 shows one intersection point in the range (0°–180°). The same intersection points are also observed and indicated by vertical lines. The angles of blue, red, and green vertical lines are about 32°, 98°, and 150°, respectively. The deviations are 66°, 52°, and 62°, respectively. This regular is also similar to the relative angular relationship (0°, 60°, and 120°) of the polarizers in Figure 1c. Therefore, the central-symmetry method performs well.

In addition, *k_ci_* in Equation (14), which is not solved by the central-symmetry method, is handled well by the calibration equation (namely, Equation (15)). When *L_S_* is 0.02 mm, *k_ci_* is between −0.5 and 0.5 mV, as shown in Figure 8. *k_i_* is between −10 and 10 mV, as shown in Table 2. Therefore, *k_ci_* is a small part of *k_i_*. The value of *L_S_* depends on the mechanical processing accuracy of the threads that fix the sensor to the rotary table. Generally, the modern mechanical processing accuracy is better than 0.01 mm. Even though *L_S_* reaches 1 mm, the central-symmetry method still offers a low ASD, as shown in Figure 12a.

The only differences between the three calibration processes, as shown in Figure 14, are the voltages used in the least-square estimation method. Note that if the number of elements of *V_inc_* in Figure 17 is too low, the estimation method cannot provide good calibration parameters, and the accuracy may be very low.

Equation (21) shows that independent of *d_a_*, the accuracy remains the same. Therefore, use of the decoupling method to obtain the calibration parameter *k_d_* does not change the accuracy. It just changes the degree of polarization slightly. In this paper, *d_a_* is slightly larger than *d_c_* to ensure that *k_d_* is negative, consistent with its physical significance, and the degree of polarization after calibration approaches the real value as close as possible.

Figure 20 shows that the values of accuracies of *θ*_12_, *θ*_13_, *θ*_23_, and *θ*_sec_ decrease from left to right. Therefore, the central-symmetry method (with the integrating sphere) and the noncontinuous method play important roles in the calibration. In the first group of columns, the accuracies of *θ*_12_, *θ*_13_, *θ*_23_, and *θ*_sec_ are similar, indicating that the section algorithm does not perform well without the central-symmetry method for indoor calibration. The central-symmetry method can be used to calibrate different types of linear polarization devices, not only the sensor used in this paper.

The theoretical-indoor replacement to process the indoor data is shown in Figure 21. When only one type of calibration parameters was replaced, the accuracies can be divided into three groups. *k_v_* and *k_d_* are in the good group. *α* is in the modest group. *k* is in the bad group. In addition, the accuracy of “*k_v_α*” or “*k_d_α*” column is the same as for “*α*” column. Similarly, the accuracy of “*kk_v_*” or “*kk_d_*” column is the same as that for “*k*” column. Therefore, it can be concluded that *k* has the strongest effect on accuracy, whereas *k_v_* or *k_d_* has a minimal effect with regard to the polarization sensor used in this paper. The “all” column shows that, without calibration, the sensor has a poor initial accuracy (±1.708°). When theoretical-outdoor2 replacement was used to process the outdoor data, see Figure 24a and Figure 25a, the same conclusion can be made as for theoretical-indoor replacement.

In addition, when Figure 24a and Figure 25a were analyzed, it was found that the indoor calibration parameters did not perform very well (a ±0.85° accuracy) in practice outdoor implications (see the green column “all” in Figure 25a). Further analysis was done to study this problem. After analyzing the good group, it was found that the accuracy of “*k_v_k_d_*” column is lower than for “*k_v_*” and “*k_d_*” columns in Figure 24a and Figure 25a, except for indoor-outdoor2 replacement. This indicates that *k_v_* and *k_d_* are closely connected and should be processed together.

For the modest group, the calibration parameters are shown in Table 2. Here, *α* for indoor, outdoor1, and outdoor2 are similar. In addition, the blue and green columns of “*α”* in Figure 24a show a similar accuracy (±0.036°), slightly larger than outdoor2 calibration accuracy (±0.014°). Therefore, the directions of linear polarizers were well calibrated for both indoor and outdoor.

For the bad group, the green columns in Figure 25a show a similar accuracy (±0.850°), indicating that indoor *k* is significantly different from outdoor2 *k*. We suggest that the indoor light source is also significantly different from the scattered skylight. In addition, the blue columns in Figure 25a show a similar accuracy, indicating that outdoor1 *k* is similar to outdoor2 *k*. Both Equations (14) and (15) show that *k* is mainly affected by the spectrum of incident light. Therefore, the spectrum of outdoor light (the scattered skylight) varies very slightly.

Not only the effects of the three groups but also the full effect of the four types of parameters was analyzed. The indoor-outdoor2 replacement values in Figure 24a and Figure 25a indicate that the indoor calibration parameters cannot be used to process outdoor data directly, even though the results are better than theoretical-outdoor2 replacement values. The blue columns in Figure 24a and Figure 25a show the highest accuracy, and the outdoor1 and outdoor2 parameters in Table 2 are very similar. Therefore, it can be concluded that the outdoor calibration parameters can be used to process the subsequently outdoor data, and a high accuracy can be obtained.

As with the analysis of Figure 24a and Figure 25a, in which the sun drift is not compensated, the same conclusion can be made when the sun drift is compensated, because Figure 24a,b are very similar and Figure 25a,b are also very similar. The similarity is an open question. During 54 s, the total sun drifts of “sun1” and “sun2” are 0.14746° and 0.13792°, respectively. If the compensation is not very precise, the accuracy after calibration will be decreased. For example, in the “sun2” calibration, when only the start point and the end point are compensated precisely by the total sun drift (0.13792°) and the medial points are compensated by linear inserting other than direct compensating, the accuracy is ±0.051°, which is much bigger than ±0.012° in Table 2.

Because the outdoor calibration and the indoor calibration obtain similar accuracies as shown in Table 2, we conclude that a clear sky and a high polarization degree is the best condition under which the sensor is used in practice application.

In the outdoor calibration process, every time before the sensor is used outdoors it needs a precise rotary table to calculate four types of parameters. This complex process should be simplified. In particular, when *k_d_* and *k_v_* are the theoretical parameters (0 and 1), α is the indoor parameter, and *k* is the outdoor parameter. The static accuracy of outdoor2 data is ±0.050°. The calculation process of the accuracy (±0.050°) is like the blue column of “all” in Figure 25a, with a combination of the theoretical, indoor, and outdoor calibration parameters as the input calibration parameters. Hence, to obtain more accurate outdoor data, we suggest that *k_d_* and *k_v_* should be the theoretical parameters; α should be the indoor parameter; and *k* must be the outdoor calibration parameter. This indicates that outdoor calibration is necessary to obtain only *k* before the measurement, which makes it easier to remove the precise rotary table in the next research work. As a result, ±0.05° is considered as the actual static accuracy when the polarization bio-sensor is used outdoors. It is better than the outdoor accuracy (between ±0.2° and ±0.42°) of the previous bio-sensors. Therefore, both the plano-convex lens and the calibration achieve substantial improvements.

The dynamic outdoor experiment shows a ±0.5° heading deviation between the polarization sensor and the inertial navigation system. The data in the two red rectangles in Figure 29 are bigger than ±0.05°. The reason might be data synchronous deviation. The polarization sensor’s frequency is decreased to 70 Hz to avoid loss of data in the transmission process. The inertial navigation system’s frequency is 10 Hz. The big angular velocities (about 30°/s) of the two turns in the experiment may cause big heading deviations when there is a little synchronous deviation, such as 0.1 s.

The deviation above (±0.5°) between the two heading measuring systems is bigger than the static experiments, even though the *k* has been replaced with the outdoor calibration parameter that is offered by outdoor calibration. There are many reasons. First, the observing direction of the polarization sensor, which is the light path as shown in Figure 1b, should coincide with the *Z*-axis of the inertial navigation system. The reference coordinate system deviation between the polarization sensor and the inertial navigation system needs a good calibration. Second, the polarization sensor should not be single. Many sensors should be used together when measuring different *E*-vectors, such as the device in the reference [20] which has five sensors. Finally, the wheel barrow, as shown in Figure 26, does not offer good shock absorption, thus the vibration is obvious.

In a future study, the blue filter will be replaced with a narrower band filter. Further optimization will be performed using the results described in this paper, which makes it possible to extend the effective range of calibration parameters while maintaining a high accuracy. New dynamic outdoor experiments, which are designed and calibrated well, will be performed.

## 7. Conclusions

A navigation bio-sensor that uses plano-convex lenses and two new calibration methods are described in this paper. The two methods are the central-symmetry calibration method (with an integrating sphere) and the noncontinuous calibration method. After calibration, the bio-sensor shows ±0.009° indoor accuracy and ±0.018° outdoor accuracy for clear-sky conditions. If more bio-sensors are used together, an even better accuracy might be obtained. In addition, the bio-sensor has a higher refresh rate (150 Hz) than the 30 Hz rate of conventional imaging polarimetry. The plano-convex lens is an essential component in this bio-sensor. The central-symmetry calibration method prominently decreased the standard deviation of voltage deviations during calibration and generated a constant value that could be easily calibrated. As a result, the redundancy of the sensor’s light path has advantages when detecting the linear polarization. The calibration parameters that were mainly determined by the spectrum of incident light dominate the accuracy, according to the analysis of calibration parameters. Before the sensor is used outdoors every time, the indoor calibration parameters *k* must be replaced with the corresponding outdoor parameters, and a static accuracy of ±0.05° can be regarded as the actual static outdoor accuracy. The dynamic outdoor experiment shows a ±0.5° heading deviation between the polarization sensor and the inertial navigation system.

## Figures and Tables

**Table 1:** Mathematical variables.

Variable	Annotation
*V* *_i_*__*ideal*_	Theoretical voltages, *i* ∈ [1, 2, 3]
*V* *_i_AD_*	Theoretical voltages of 16-bit ADC, *i* ∈ [1, 2, 3]
*V_ref_*	Reference voltage for logarithmic amplifier
*V_i_*	Voltages, *i* ∈ [1, 2, 3]
*V* *_imr_*	Voltages obtained using the central-symmetry method, also mean values of *V_ibr_* and *V_i_*_a*r*_, *i* ∈ [1, 2, 3]
*V* *_inc_*	Voltages obtained using the noncontinuous method, *i* ∈ [1, 2, 3]
*V_br_*	Voltage before 180° rotation
*V*_a*r*_	Voltage after 180° rotation
V*_icf_*	Theoretical voltages calculated by the calibration parameters in Equation (21)
*θ*	Polarization angle
*θ*_12_, *θ*_13_, *θ*_23_	Polarization angles from three voltages
*θ_R_*	Rotational angle of precise rotary table in simulation
*θ*_sec_	Polarization angle obtained using the section algorithm
*d*	Polarization degree
*d_c_* *_m_*	Polarization degree obtained using the iterative least-squares estimation method, *m* ∈ [1, 2, 3, 4, 5, 6]
*d_a_*	Polarization degree determined by the authors
*O_IS_*	Center of integrating sphere
*O_S_*	Center of polarization sensor
*O_R_*	Center of rotary table
*L* *_o_*	Length between the port and the photosensitive surface
*E_o_*	Irradiance at the center
*E_e_*	Irradiance at the off-axis edge
*E*_1_, *E*_2_, *E’*_1_, *E’*_2_	Irradiance at Point 1, 2, 1’, 2’
*L* *_photo_*	Length between *O_S_* and the photosensitive surface
*α_IS_*	Eccentric angle between *O_R_* and *O_IS_*
*α_S_*	Eccentric angle between *O_R_* and *O_S_*
*L* *_IS_*	Eccentric distance between *O_R_* and *O_IS_*
*L* *_S_*	Eccentric distance between *O_R_* and *O_S_*
*L* *_R_*_1_	Eccentric distance between *O_R_* and Point 1
*L* *_R_*_2_	Eccentric distance between *O_R_* and Point 2
*L*_1_, *L*_2_, *L*_1_’, *L*_2_’	Off-axis distance between *O_IS_* and Point 1, 2, 1’, 2’
BSD	Standard deviation of a 360° range before the central-symmetry method is used
ASD	Standard deviation of a 360° range after the central-symmetry method is used
*g_up_*	Gain of unpolarized light
*g_tp_*	Gain of totally polarized light
*τ* *_M_*	Transmittance when the reference angle and main polarization angle of incident light are parallel
*τ* *_m_*	Transmittance when the reference angle and main polarization angle of incident light are orthogonal
*τ* *_f_*	Transmittance of blue filter
*E_in_*	Irradiance of incident light
*E* *_p_*	Irradiance at photodiode
*s_r_*	Spectral responsivity of photodiode
*A_r_*	Active area size of photodiode
*k* *_ci_*	Constant value generated by the integrating sphere method, *i* ∈ [1, 2, 3]
*k* *_i_*	Additive coefficient of calibration, *i* ∈ [1, 2, 3]
*k_v_* *_i_*	Deviation parameter of reference voltage of logarithmic amplifier, *i* ∈ [1, 2, 3]
*k* *_dm_*	Coefficient of non-ideal polarizer, *m* ∈ [1, 2, 3, 4, 5, 6]
*α* *_m_*	Installation angles of polarizer, *m* ∈ [1, 2, 3, 4, 5, 6]

**Table 2:** Calibration parameters.

Parameter	Theory	Indoor	Outdoor1	Outdoor2	Sun1	Sun2
α_1_ (°)	0	0.7554	0.7813	0.9189	0.7887	0.9344
α_2_ (°)	0	0.5500	0.8017	0.4385	0.8236	0.4540
α_3_ (°)	−120	−117.6462	−117.8128	−118.0658	−117.7964	−118.0503
α_4_ (°)	−120	−116.5699	−116.3585	−116.3233	−116.3421	−116.3078
α_5_ (°)	120	123.0730	123.3667	122.9320	123.3727	122.9510
α_6_ (°)	120	120.6994	120.6410	120.6542	120.6634	120.6634
*k*_1_ (mV)	0	−10.1519	−7.4487	−7.8456	−7.4461	−7.8455
*k*_2_ (mV)	0	7.9230	8.8675	9.3682	8.8674	9.3681
*k*_3_ (mV)	0	−9.4542	−18.5845	−18.7583	−18.5778	−18.7604
*k_d_*_1_	0	−0.0438	−0.0229	−0.0277	−0.0203	−0.0246
*k_d_*_2_	0	−0.0365	−0.0074	−0.0153	−0.0100	−0.0158
*k_d_*_3_	0	−0.0457	−0.0118	−0.0050	−0.0127	−0.0086
*k_d_*_4_	0	−0.0492	−0.0255	−0.0163	−0.0217	−0.0166
*k_d_*_5_	0	−0.0555	−0.0151	−0.0179	−0.0136	−0.0140
*k_d_*_6_	0	−0.0166	−0.0137	−0.0217	−0.0134	−0.0172
*k_v_*_1_	1	1.0224	1.0064	1.0119	1.0066	1.0119
*k_v_*_2_	1	1.0081	1.0135	1.0029	1.0135	1.0029
*k_v_*_3_	1	1.0183	1.0070	1.0125	1.0054	1.0073
*d*_a_	-----	0.5500	0.6550	0.7100	0.6600	0.7150
Origin-Accuracy (°)	-----	±0.0088	±0.0177	±0.0140	±0.0207	±0.0119
Accuracy (°)	-----	±0.009	±0.018	±0.014	±0.021	±0.012

## References

[1] Cochran W.W., Mouritsen H., Wikelski M. (2004). Migrating songbirds recalibrate their magnetic compass daily from twilight cues. Science.

[2] Muheim R., Phillips J.B., Akesson S. (2006). Polarized light cues underlie compass calibration in migratory songbirds. Science.

[3] Daly I.M., How M.J., Partridge J.C., Temple S.E., Marshall N.J., Cronin T.W., Roberts N.W. (2016). Dynamic polarization vision in mantis shrimps. Nat. Commun..

[4] Schwarz S., Mangan M., Zeil J., Webb B., Wystrach A. (2017). How Ants Use Vision When Homing Backward. Curr. Biol..

[5] Lambrinos D., Moller R., Labhart T., Pfeifer R., Wehner R. (2000). A mobile robot employing insect strategies for navigation. Rob. Auton. Syst..

[6] Chu J., Zhao K. (2005). Study of Angle Measure Optoelectronic Model on Emulating Polarization-Sensitive Compound Eye of Insect. Nanoelectron. Device Technol..

[7] Chu J., Zhao K., Zhang Q., Wang T. (2008). Construction and performance test of a novel polarization sensor for navigation. Sens. Actuators A.

[8] Labhart T., Meyer E.P. (1999). Detectors for polarized skylight in insects: A survey of ommatidial specializations in the dorsal rim area of the compound eye. Microsc. Res. Techniq..

[9] Xian Z., Hu X., Lian J., Zhang L., Cao J., Wang Y., Ma T. (2014). A Novel Angle Computation and Calibration Algorithm of Bio-Inspired Sky-Light Polarization Navigation Sensor. Sensors.

[10] Ma T., Hu X., Zhang L., He X. (2016). Calibration of a polarization navigation sensor using the NSGA-II algorithm. Opt. Commun..

[11] Zhao H., Xu W. (2016). A Bionic Polarization Navigation Sensor and Its Calibration Method. Sensors.

[12] Yang J., Du T., Niu B., Li C., Qian J., Guo L. (2018). A Bionic Polarization Navigation Sensor Based on Polarizing Beam Splitter. IEEE Access.

[13] Dupeyroux J., Diperi J., Boyron M., Viollet S., Serres J. A novel insect-inspired optical compass sensor for a hexapod walking robot. Proceedings of the IEEE International Conference on Intelligent Robots and Systems.

[14] Dupeyroux J., Viollet S., Serres J. (2019). Polarized skylight-based heading measurements: A bio-inspired approach. J. R. Soc. Interface.

[15] Dupeyroux J., Serres J., Viollet S. (2019). AntBot: A six-legged walking robot able to home like desert ants in outdoor environments. Sci. Robot..

[16] Du T., Li X., Wang Y., Yang J., Liu W. (2019). Multiple Disturbance Analysis and Calibration of an Inspired Polarization Sensor. IEEE Access.

[17] Serres J.R., Viollet S. (2018). Insect-inspired vision for autonomous vehicles. Curr. Opin. Insect. Sci..

[18] Suhai B., Horvath G. (2004). How well does the Rayleigh model describe the E-vector distribution of skylight in clear and cloudy conditions? A full-sky polarimetric study. J. Opt. Soc. Am. A.

[19] Wang Y., Chu J., Zhang R., Wang L., Wang Z. (2015). A novel autonomous real-time position method based on polarized light and geomagnetic field. Sci. Rep..

[20] Wang Y., Chu J., Zhang R., Shi C. (2018). Orthogonal vector algorithm to obtain the solar vector using the single-scattering Rayleigh model. Appl. Opt..

[21] Zhi W., Chu J., Li J., Wang Y. (2018). A Novel Attitude Determination System Aided by Polarization Sensor. Sensors.

[22] Szaz D., Farkas A., Barta A., Kretzer B., Blaho M., Egri A., Szabo G., Horvath G. (2017). Accuracy of the hypothetical sky-polarimetric Viking navigation versus sky conditions: Revealing solar elevations and cloudinesses favourable for this navigation method. Proc. R. Soc. A Math. Phys. Eng. Sci..

[23] Horvath G., Takacs P., Kretzer B., Szilasi S., Szaz D., Farkas A., Barta A. (2017). Celestial polarization patterns sufficient for Viking navigation with the naked eye: Detectability of Haidinger’s brushes on the sky versus meteorological conditions. R. Soc. Open Sci..

[24] Száz D., Horváth G. (2018). Success of sky-polarimetric Viking navigation: Revealing the chance Viking sailors could reach Greenland from Norway. R. Soc. Open Sci..

[25] Lu H., Zhao K., You Z., Huang K. (2015). Angle algorithm based on Hough transform for imaging polarization navigation sensor. Opt. Express.

[26] Fan C., Hu X., Lian J., Zhang L., He X. (2016). Design and Calibration of a Novel Camera-Based Bio-Inspired Polarization Navigation Sensor. IEEE Sens. J..

[27] Lu H., Zhao K., You Z., Huang K. (2017). Real-time polarization imaging algorithm for camera-based polarization navigation sensors. Appl. Opt..

[28] Zhang W., Cao Y., Zhang X., Yang Y., Ning Y. (2017). Angle of sky light polarization derived from digital images of the sky under various conditions. Appl. Opt..

[29] Fan C., Hu X., He X., Zhang L., Lian J. (2018). Integrated Polarized Skylight Sensor and MIMU With a Metric Map for Urban Ground Navigation. IEEE Sens. J..

[30] Chu J., Lin L., Chen W., Wang Y. (2012). Design and realization of bionic polarized light navigation sensor based on MSP430. Transducer Microsyst. Technol..

[31] Pust N.J., Shaw J.A. (2008). Digital all-sky polarization imaging of partly cloudy skies. Appl. Opt..

[32] Tudor T., Manea V. (2011). Symmetry between partially polarised light and partial polarisers in the vectorial Pauli algebraic formalism. J. Mod. Optic..

